# *Brevundimonas brasiliensis* sp. nov.: a New Multidrug-Resistant Species Isolated from a Patient in Brazil

**DOI:** 10.1128/spectrum.04415-22

**Published:** 2023-04-17

**Authors:** Gabriela Guerrera Soares, Emeline Boni Campanini, Roumayne Lopes Ferreira, Marcelo Silva Folhas Damas, Saulo Henrique Rodrigues, Leslie Camelo Campos, Jucimária Dantas Galvão, Andrea Soares da Costa Fuentes, Caio César de Melo Freire, Iran Malavazi, André Pitondo-Silva, Anderson Ferreira da Cunha, Maria-Cristina da Silva Pranchevicius

**Affiliations:** a Departamento de Genética e Evolução, Universidade Federal de São Carlos, São Carlos, São Paulo, Brazil; b Laboratório Central de Saúde Pública do Tocantins, Palmas, Tocantins, Brazil; c Programas de Pós-graduação em Odontologia e Tecnologia Ambiental, Universidade de Ribeirão Preto, Ribeirão Preto, São Paulo, Brazil; d Centro de Ciências Biológicas e da Saúde, Biodiversidade Tropical - BIOTROP, Universidade Federal de São Carlos, São Carlos, São Paulo, Brazil; Public Health Ontario

**Keywords:** *Brevundimonas brasiliensis* sp. nov., multidrug resistance profile, whole-genome sequencing

## Abstract

To increase knowledge on *Brevundimonas* pathogens, we conducted in-depth genomic and phenotypic characterization of a *Brevundimonas* strain isolated from the cerebrospinal fluid of a patient admitted in a neonatal intensive care unit. The strain was identified as a member of the genus *Brevundimonas* based on Vitek 2 system results and 16S rRNA gene sequencing and presented a multidrug resistance profile (MDR). Several molecular and biochemical tests were used to characterize and identify the species for in-depth results. The draft genome assembly of the isolate has a total length of 3,261,074 bp and a G+C of 66.86%, similar to other species of the genus. Multilocus sequence analysis, Type (Strain) Genome Server, digital DNA-DNA hybridization, and average nucleotide identity confirmed that the *Brevundimonas* sp. studied represents a distinct species, for which we propose the name *Brevundimonas brasiliensis* sp. nov. *In silico* analysis detected antimicrobial resistance genes (AMRGs) mediating resistance to β-lactams (*penP*, *bla*_TEM-16_, and *bla*_BKC-1_) and aminoglycosides [*strA*, *strB*, *aac(6′)-Ib*, and *aac(6′)-Il*]. We also found AMRGs encoding the AcrAB efflux pump that confers resistance to a broad spectrum of antibiotics. Colistin and quinolone resistance can be attributed to mutation in *qseC* and/or *phoP* and GyrA/GyrB, respectively. The *Brevundimonas brasiliensis* sp. nov. genome contained copies of type IV secretion system (T4SS)-type integrative and conjugative elements (ICEs); integrative mobilizable elements (IME); and Tn*3*-type and IS*3*, IS*6*, IS*5*, and IS*1380* families, suggesting an important role in the development and dissemination of antibiotic resistance. The isolate presented a range of virulence-associated genes related to biofilm formation, adhesion, and invasion that can be relevant for its pathogenicity. Our findings provide a wealth of data to hinder the transmission of MDR *Brevundimonas* and highlight the need for monitoring and identifying new bacterial species in hospital environments.

**IMPORTANCE** *Brevundimonas* species is considered an opportunistic human pathogen that can cause multiple types of invasive and severe infections in patients with underlying pathologies. Treatment of these pathogens has become a major challenge because many isolates are resistant to most antibiotics used in clinical practice. Furthermore, there are no consistent therapeutic results demonstrating the efficacy of antibacterial agents. Although considered a rare pathogen, recent studies have provided evidence of the emergence of *Brevundimonas* in clinical settings. Hence, we identified a novel pathogenic bacterium, *Brevundimonas brasiliensis* sp. nov., that presented a multidrug resistance (MDR) profile and carried diverse genes related to drug resistance, virulence, and mobile genetic elements. Such data can serve as a baseline for understanding the genomic diversity, adaptation, evolution, and pathogenicity of MDR *Brevundimonas.*

## INTRODUCTION

*Brevundimonas* spp. are aerobic Gram-negative, nonfermenting rods, belonging to the *Caulobacteraceae* family (1). Usually, they are isolated from various environments, such as purified water (2), deep subsea floor sediment (3, 4), black sand (5), and soils (6, 7). Although these species might be part of human microbiota, the role that they play in human patients without significant diseases has not been determined (8, 9).

Since *Brevundimonas* spp. are considered to be opportunistic human pathogens in immunocompromised patients (10), they can cause multiple types of infections, including septicemia (11), pneumonia/pleuritis (12), endocarditis (13, 14), meningitis (15), keratitis (16), and urinary tract infections (17, 18). To date, there are 34 species with a valid species published with a correct name within the genus *Brevundimonas* (http://www.bacterio.net/brevundimonas.html). New species belonging to the genus *Brevundimonas* have been isolated from a variety of sources, including the feces of a patient with diarrhea (*Brevundimonas pishanensis* sp. nov.) (19) and the blood of a patient with hepatocellular carcinoma (*Brevundimonas huaxiensis*) (20).

Whole-genome sequencing (WGS) has become a valuable tool for enhancing diagnostic, public health surveillance, and antimicrobial drug resistance (21). It can also be used for identifying novel and rare species and resolving inconsistencies among commonly used methods of bacterial identification, such as 16S rRNA sequence analysis and phenotyping (22). Average nucleotide identity (ANI) and digital DNA-DNA hybridization (dDDH) are WGS-based experimental methods developed to determine the similarity between two genomes (23) and have been widely used as a gold standard for species delineation (24).

In this study, a multidrug-resistant (MDR) *Brevundimonas* sp., isolated from a cerebrospinal fluid sample of a hospitalized infant, could not be identified by the Vitek 2 system or by 16S rRNA sequence analysis. Thus, we identified a novel species, *Brevundimonas brasiliensis* sp. nov., using traditional methods of bacterial identification and multiple comparative genomic analyses. Moreover, the draft genome of *Brevundimonas brasiliensis* sp. nov. was subjected to an in-depth analysis of genes related to the pathogenicity, antimicrobial resistance, and mobile genetic elements.

## RESULTS

### Bacteria isolation and antibiotic susceptibility profile.

The bacterial isolate was correctly identified at the genus level as a *Brevundimonas* sp. using the Vitek 2 system. However, this method was not accurate for species-level identification. Antibiotic susceptibility was also performed using the Vitek 2 system, and the isolate was classified as MDR as it was nonsusceptible to at least three different antimicrobial classes, including beta-lactams (ampicillin, ampicillin/sulbactam, piperacillin-tazobactam, cefuroxime axetil, cefoxitin, ceftazidime, ceftriaxone, cefepime, ertapenem, imipenem, and meropenem); aminoglycosides (amikacin and gentamicin); polymyxin (colistin); and quinolone (ciprofloxacin) (Table 1). The *Brevundimonas* sp. sample showed susceptibility to only tigecycline, an antibiotic belonging to the glycylcycline class.

**TABLE 1:** Resistome of *Brevundimonas brasiliensis* sp. nov.

Phenotypic antibiotic class	Phenotypic antibiotic resistance (Vitek 2)^*a*^	Genotyping/properties of proteins					
		Reference sequence (NCBI and KEGG)	Putative resistance genes	Resistance gene/protein, mechanism function	Size (aa)	aa identity (%)	Resistance gene characterization
Beta-lactams	Ampicillin, ampicillin-sulbactam, piperacillin-tazobactam, cefuroxime axetil, cefoxitin, ceftazidime, ceftriaxone, cefepime, ertapenem, imipenem, meropenem	K17836	*penP*	Beta-lactamase class A	299	60.00	KEGG
		U36911	*bla*_TEM-116_	Class A broad-spectrum beta-lactamase TEM-116	286	100.00	AMRFinderPlus; RESfinder; ARG-ANNOT; CARDonline; CARDblast; AMRfinder; Bacant
		KP689347	*bla*_BKC-1_	BKC family carbapenem-hydrolyzing class A beta-lactamase	605	100.00	RESfinder; ARG-ANNOT; CARDblast; AMRfinder; Bacant
Aminoglycosides	Amikacin, Gentamicin	AB366441	*strA*	Streptomycin 3′-kinase	139	100.00	ARG-ANNOT; KEGG
		FJ474091	*strB*	Streptomycin 6-kinase	279	100.00	ARG-ANNOT; KEGG
		AJ584652	*aac(6′)-Ib'*	Aminoglycoside N-acetyltransferase AAC(6′)-Ib'	210	100.00	CARDblast; AMRfinder; Bacant
		U13880	*aac(6′)-Il*	Aminoglicosídeo N-acetiltransferase AAC(6′)-Il	459	100.00	RESfinder; CARDonline
Quinolones	Ciprofloxacin	ADL01935	*gyrA* ^*b*^	DNA gyrase subunit A	924	86.00	Prokka; RAST; KEGG
		ADK99321	*gyrB* ^*b*^	DNA gyrase subunit B	809	84.00	Prokka; RAST; KEGG
		EU370913	*oqxBgb*	OqxB integral membrane protein	396	48.20	ARG-ANNOT
Polymyxin	Colistin	ALJ07207	*qseC* ^*b*^	Two-component system, OmpR family, sensor histidine kinase QseC	439	61.00	Prokka; KEGG
			*phoP* ^*b*^	DNA-binding transcriptional dual regulator PhoP	226		Prokka
Efflux pump (Chloramphenicol, fluoroquinolone, tetracycline, novobiocin, fusidic acid, nalidixic acid, β-lactam antibiotics, quinolones, β-lactams, tetracycline, chloramphenicol, macrolides, triclosan resistance)	(−)	ADL00797	*acrA* like	Multidrug efflux pump membrane fusion lipoprotein AcrA	381	66.00	KEGG
		ADL00798	*acrB* like	Multidrug efflux pump RND permease AcrB	1,079	83.00	KEGG
		ADL01129	*tolC* like	Outer membrane channel TolC	524	65.00	KEGG
		AKH42737	*oprM*	Outer membrane protein OprM	495	99.00	KEGG
		ADK99730	*mexL*	Regulatory protein TetR	199	63.00	KEGG
Tetracycline	(−)	AAN06707.1	*tet(A)*	Tetracycline resistance protein, class A Tet(A)	425	77.00	KEGG
Glycopeptide	(−)	ADL01677	*vanY*	VanY-A/VanY-F/VanY-M family D-Ala-D-Ala carboxypeptidase	285	66	KEGG
Sulfonamide	(−)	JF969163	*sul1*	Sulfonamide-resistant dihydropteroate synthase Sul1	279	100.00	RESfinder; ARG-ANNOT; CARDonline; CARDblast; AMRfinder; Bacant
Cationic antimicrobial peptide resistance	(−)	Q8FW76	*mprF*	Bifunctional lysyl phosphatidylglycerol flippase/synthetase MprF	554	73	KEGG
Phenicol	(−)	AKLJ01000508	*floR*	Chloramphenicol/florfenicol efflux MFS transporter FloR;	286	95.122	RESfinder; ARG-ANNOT; CARDblast; AMRfinder; Bacant
Diaminopyrimidine trimethoprim	(−)	AY552589	*dfrA21*	Trimethoprim-resistant dihydrofolate reductase type A21	165	100.00	RESfinder; ARG-ANNOT; CARDonline; CARDblast; AMRfinder; Bacant

a (−), Susceptibility testing was not performed.

b Mutations in genes conferring resistance to quinolones (GyrA, S83L and D87H; GyrB, Leu-466) and colistin (PhoP, Arg81Cis and *qse*C, Ile283Leu).

### Features of the genome assembly.

**FIG 1:**
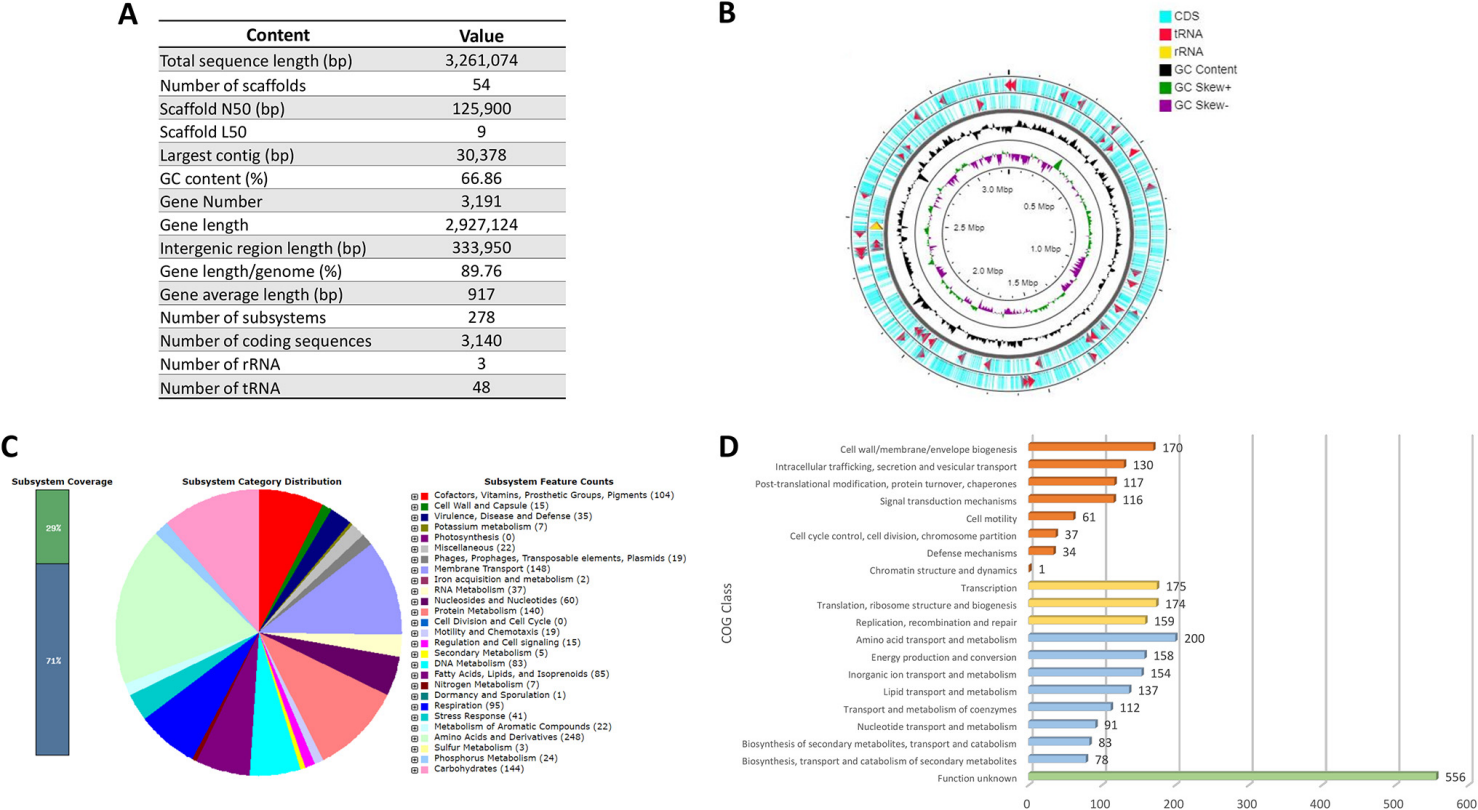
(A) Genome statistics of *Brevundimonas* sp. (B) A circular representation of the chromosome of *Brevundimonas* sp. The outermost and the second circle show the coding sequence (CDS), tRNA, and rRNA. The third circle shows the GC content (black). The fourth circle demonstrates the GC skew curve (positive GC skew, green; negative GC skew, violet). Markings inside the innermost circle represent genome positions in Mbp. (C) Subsystem category distribution of major protein coding genes by the RAST annotation server. The bar chart shows the subsystem coverage in percentage, in which the green bar corresponds to the percentage of proteins included. The pie chart shows the percentage distribution of the 27 subsystem categories. (D) COG classification in the following four main categories: metabolism (blue bars), information processing and storage (yellow), cellular processes and signaling (red bars), and unknown function (green bar).

The draft genome size of the *Brevundimonas* sp. comprised 3,261,074 bp, a G+C content of 66.86%, 54 scaffolds, and 3,191 genes that covered 89.76% of the genome. Among these genes, 3,140 were coding sequences (CDSs), and 51 were noncoding RNAs (including 48 tRNAs and 3 rRNAs) (Fig. 1A and B). A genome analysis of *Brevundimonas* sp. by the RAST Server revealed 278 subsystems classified into 27 categories. Among these categories, the “amino acid and derivatives” subsystem comprised the largest number (248 CDSs), followed by “membrane transport” (148), “carbohydrates” (144), and “protein metabolism” (140). The “virulence, disease, and defense” subsystem accounted for 35 CDSs, with 21 of them associated with resistance to antibiotics and toxic compounds (16 copper homeostasis, 2 resistance to fluoroquinolones, 1 beta-lactamase, 1 multidrug resistance efflux pump, 1 copper tolerance), while 14 were related to invasion and intracellular resistance (Fig. 1C). The data of whole-genome sequencing, circular representations, and subsystem category distributions are shown in Fig. 1.

The distribution of protein-coding genes into the cluster of orthologous groups (COG) functional category showed a total of 2,743 genes (Fig. 1D). The majority of known protein-coding genes were associated with “metabolism” (*n* = 1,013; 36.93%), followed by those related to “cellular processes and signaling” (*n* = 666; 24.28%), and “information storage and processing” (*n* = 508; 18.51%). The number of genes associated with “unknown functions” was 556 (20.26%) and with defense was 34 (1.23%) (Fig. 1D).

### Phylogenetic tree and biochemical analysis.

**FIG 2:**
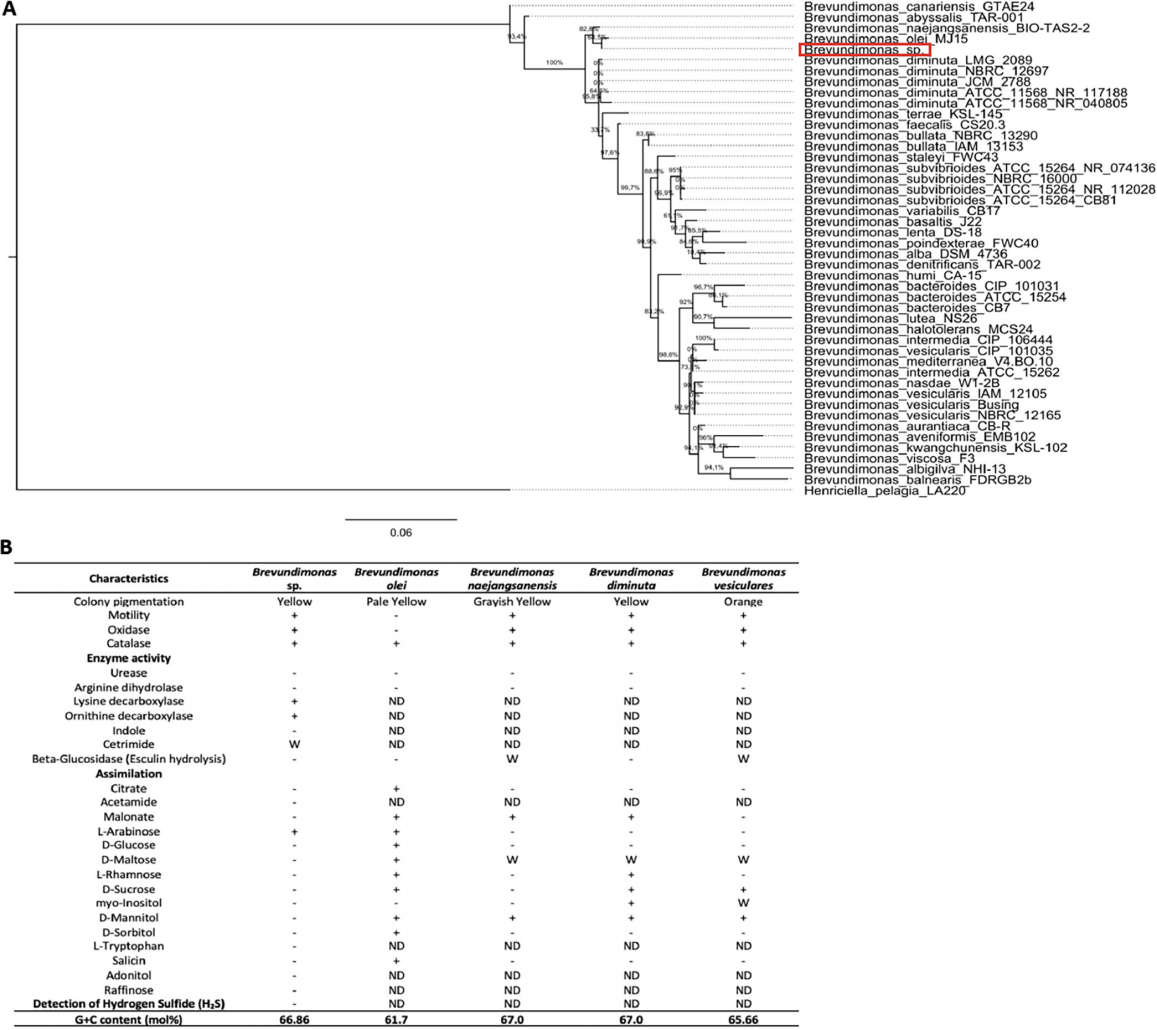
(A) Maximum-likelihood tree based on 16S rRNA gene sequences of strain *Brevundimonas* sp. and the members of the genus *Brevundimonas*. (B) Phenotypic characteristics that differentiate strain *Brevundimonas* sp. from some related *Brevundimonas* species. Data for *Brevundimonas* sp. are from this study, and data for *B. olei* MJ15^T^, B. naejangsanensis KCTC 22631^T^, *B. diminuta* KCTC 12488^T^, and *B. vesicularis* KCTC 12421^T^ are from reference 146. +, positive; −, negative; W, weak reaction; ND, no data available.

The genomic sequence of *Brevundimonas* sp. presented only one 16S rRNA gene sequence, indicating that the genome assembly was not contaminated by other organisms. Therefore, a phylogenetic tree was constructed based on the 16S rRNA gene sequence (1,459 bp) of our strain and all 16S rRNA gene sequences (*n* = 44) of known *Brevundimonas* species deposited in GenBank. The 16S rRNA reference sequence of Henriciella pelagia strain LA220 was used as an outgroup. The results confirmed that the *Brevundimonas* sp. represents a member of the genus *Brevundimonas*. In this initial taxonomic classification, *Brevundimonas* sp. was most related to *Brevundimonas olei* with a sequence identity of 99.71% (with 68.5% bootstrap support), followed by Brevundimonas naejangsanensis (BIO TAS2-2) (Fig. 2A). To define the characteristics of *Brevundimonas* sp., biochemical tests were performed and compared with *Brevundimonas olei*, Brevundimonas naejangsanensis, Brevundimonas diminuta, and Brevundimonas vesicularis (Fig. 2B). Unlike *B. olei*, our strain was oxidase positive and motile. The results also showed that *Brevundimonas* sp. had a yellow color, it was catalase positive, and it only assimilated l-arabinose. The nonutilization of d-mannitol is unique to our strain when compared with other *Brevundimonas* species (Fig. 2B).

### Genetic relatedness.

**FIG 3:**
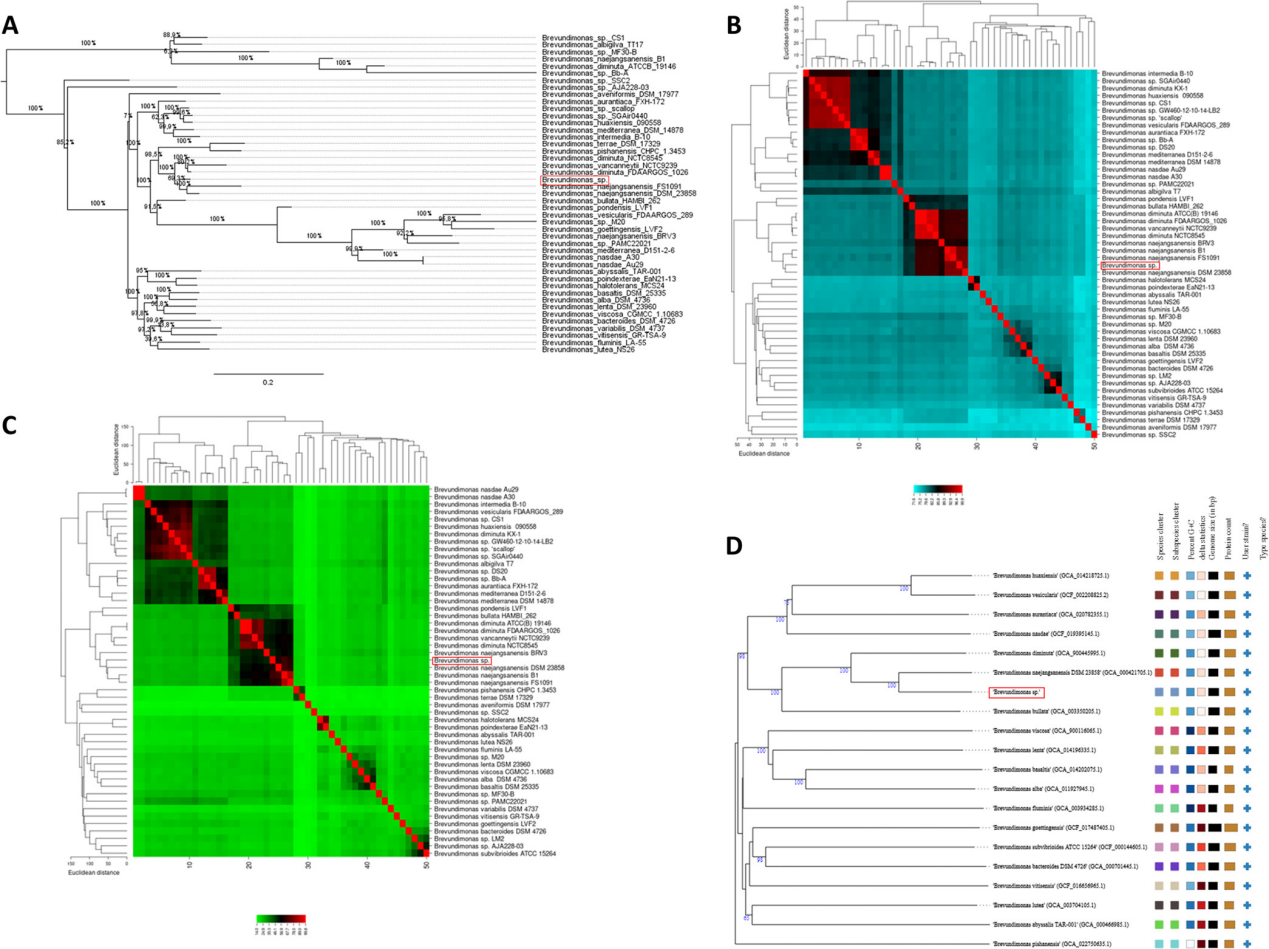
Comparative genomic analysis of *Brevundimonas* spp. (A) MLSA phylogenetic tree. (B) Heatmap with ANI results. (C) Heatmap with calculations referring to dDDH. (D) Phylogenomic tree based on TYGS results showing the relationship between *Brevundimonas* sp. strain with *Brevundimonas* reference sequence strains deposited at NCBI. Tree inferred with FastME 2.1.6.1 (147) from GBDP (BLAST genome distance phylogeny method) distances calculated from genomic sequences. Branch lengths are scaled in terms of the GBDP distance formula d5. The numbers above the branches are GBDP pseudobootstrap support values > 60% of 100 replications, with an average branch support of 75.1%. The tree was rooted at the midpoint (148).

To further determine the taxonomic affiliation of *Brevundimonas* sp., a multilocus sequence analysis (MLSA) was performed with five housekeeping genes found in complete genomic and reference sequences of *Brevundimonas* (see Table S3 in the supplemental material). The phylogenetic trees (Fig. 3A) were generated based on the concatenated sequences in the following order: *atpD* (1,536 bp), *recA* (1,080 bp), *ileS* (2,922 bp), *rpoD* (1,923 bp), and *trpB* (1,224 bp), which yielded an alignment of 8,684 bp. The MLSA tree exhibited the close association between our *Brevundimonas* sp. and Brevundimonas naejangsanensis FS1091 (Fig. 3A), followed by Brevundimonas naejangsanensis DSM 23858.

To clarify the results of MLSA, ANI and digital DNA-DNA hybridization (dDDH) analyses were performed with the *Brevundimonas* sp. and all 49 complete genomic and 27 reference sequences from the genus *Brevundimonas* available in GenBank (see Table S4 in the supplemental material). In these analyses, the closest fully assembled genome were Brevundimonas naejangsanensis DSM 23858 (ANI = 93.15%, dDDH = 63.0%) followed by Brevundimonas naejangsanensis B1 (ANI = 92.60%, dDDH = 67.3%), Brevundimonas naejangsanensis FS1091 (ANI = 92.52%, dDDH = 62.9%), and Brevundimonas vancanneytii NCTC9239 (ANI = 89.31%, dDDH = 54.5%) (Fig. 3B and C). Based on these findings, *Brevundimonas* sp. represents a novel species (ANI < 95%, dDDH < 70%).

A phylogenetic tree based on 19 reference genome sequences and the *Brevundimonas* sp. was constructed using Type (Strain) Genome Server (TYGS). The TYGS-based results showed that *Brevundimonas* sp. are most closely related to Brevundimonas naejangsanensis DSM 23858 (Fig. 3D), with dDDH values (formula *d*4) of 50.8%, also positioning *Brevundimonas* sp. as a novel species.

Although *Brevundimonas olei* presented >99% 16S rRNA sequence identity with the *Brevundimonas* sp., it had no housekeeping genes or genome sequence available in genetic sequence database for comparison. Therefore, *Brevundimonas olei* was not included in MLSA, ANI, dDDH, or TYGS analysis.

### Genome properties and comparative functional analysis.

**FIG 4:**
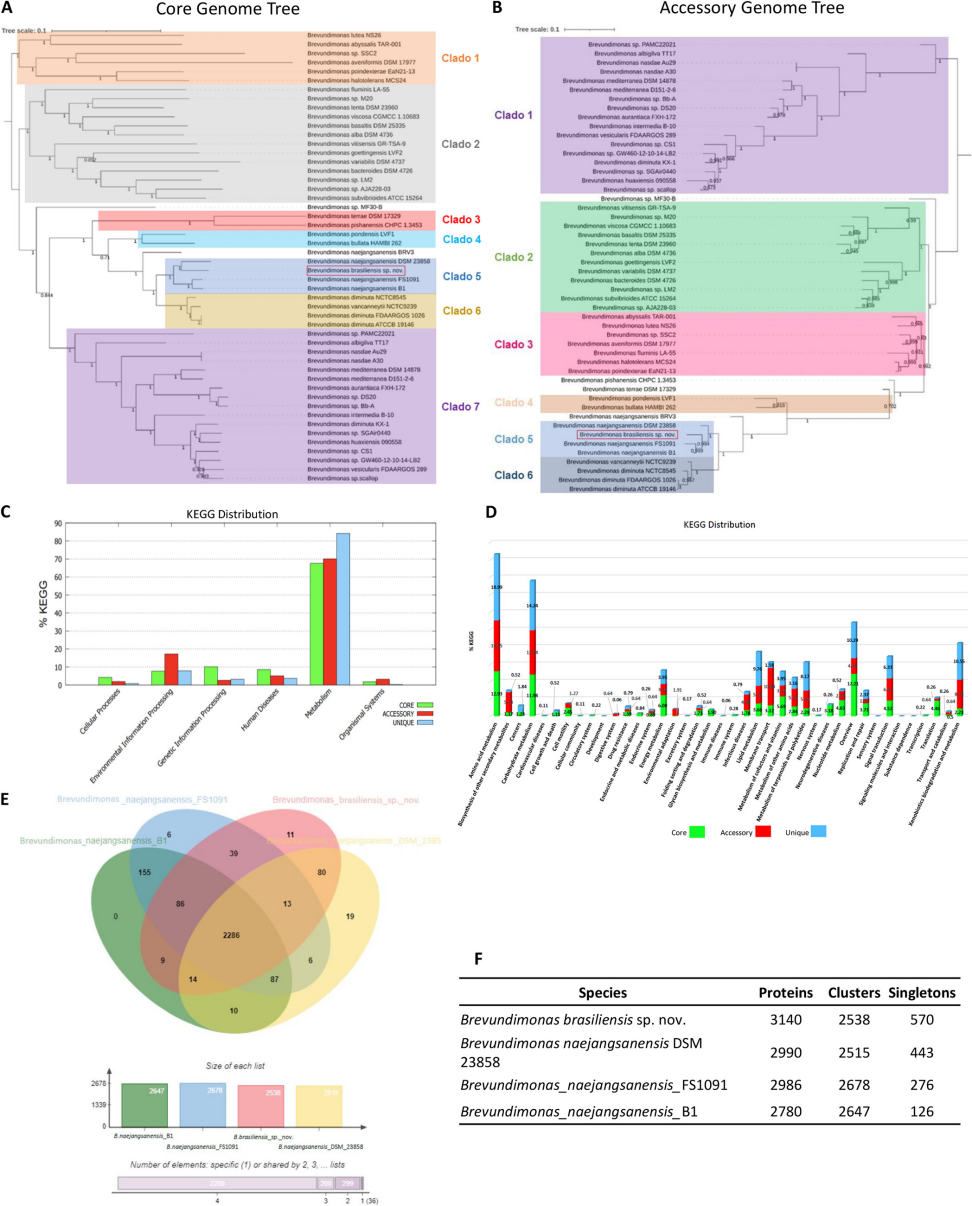
Comparative genomic analysis. (A) Phylogenetic tree constructed using the core genome. (B) Phylogenetic tree constructed using the accessory genome. (C) KEGG pathway classification in core, accessory, and unique genomes in the four most closely related *Brevundimonas* species (clade 5). (D) Distribution of KEGG pathway classification. (E) Venn diagram and bar graph showing the numbers of unique and shared orthologous genes present in *Brevundimonas* spp. (clade 5). (F) Number of proteins, clusters, and singletons.

To investigate general evolutionary patterns of genomes, we constructed two phylogenetic trees based on the set of core and accessory genomes of our strain with 49 reference and complete genomes of *Brevundimonas* deposited in GenBank. The trees were divided into seven main clusters, according to topological structure and evolutionary distance. The relative positions of *Brevundimonas brasiliensis* sp. nov., Brevundimonas naejangsanensis DSM 23858, Brevundimonas naejangsanensis FS1091, and Brevundimonas naejangsanensis B1 species (clade 5) varied between the two trees. *Brevundimonas brasiliensis* sp. nov. and Brevundimonas naejangsanensis DSM 23858 were segregated under a common node in the core genome tree, although the strains segregated together under distinct nodes in the accessory genome tree (Fig. 4A and B).

Next, we compared the gene functional distribution among the four species present in clade 5 (Fig. 4C and D). The KEGG analysis revealed that 77% (*n* = 1,794) of genes were associated with core, 16.3% (*n* = 379) with unique, and 6.7% (*n* = 157) with accessory. Genes associated with “metabolism” accounted for the largest proportion both in the unique (84.16%; *n* = 319), accessory (70.06%; *n* = 110), and core (67.61%; *n* = 1,213) genomes. The annotations of these unique genes indicated that most were related to “amino acid metabolism” (18.99%; *n* = 72), “carbohydrate metabolism” (14.24%; *n* = 54), and xenobiotics biodegradation and metabolism (10.55%; *n* = 40). The core genes were mainly related to “amino acid metabolism” (12.93%; *n* = 232), overview (12.21%; *n* = 219), and “carbohydrate metabolism” (11.98%; *n* = 215). The accessory genes were associated with “amino acid metabolism” (14.65%; *n* = 23) and “carbohydrate metabolism” (12.74%; *n* = 20). The genes involved in “human diseases” (*n* = 176; 7.54%) accounted for 6.6% (*n* = 154) in core, 0.6% (*n* = 14) in unique, and 0.34% (*n* = 8) in accessory. Interestingly, we found genes related to beta-lactam (5.68%; *n* = 10), vancomycin (2.84%; *n* = 5), and cationic antimicrobial peptide (CAMP) resistance (5.68%; *n* = 10) in core gene clusters for human disease.

Orthologous gene cluster analysis was also performed among the *B. brasiliensis* sp. nov. and the Brevundimonas naejangsanensis DSM 23858, *B. naejangsanensis* FS1091, and *B. naejangsanensis* B1 strains. The four strains shared 1,661 orthologous gene clusters. *B. brasiliensis* sp. nov. shared 80 orthologous gene clusters with B*. naejangsanensis* DSM 23858, 39 with *B. naejangsanensis* strain FS1091, and 9 with *B. naejangsanensis* B1. The number of unique orthologue gene clusters was 11 for the *B. brasiliensis* sp., 19 for *B. naejangsanensis* DSM 23858, 6 for *B. naejangsanensis* FS1091, and none for *B. naejangsanensis* B1. The Venn diagram orthologous gene clusters distributed among the four strains is shown in Fig. 4E. *Brevundimonas brasiliensis* sp. nov. strain contained the highest number of singletons (*n* = 570), followed by *B. naejangsanensis* DSM 23858 (*n* = 443), *B. naejangsanensis* strain FS1091 (*n* = 276), and *B. naejangsanensis* B1 (*n* = 126) (Fig. 4F). The singletons from *B. brasiliensis* sp. presented *strA*, *strB*, *aac(6')-Ib*, and *drfA21* genes related to antibiotic resistance and *virB11*, *cheB*, *mrsA/B* genes associated with virulence.

### Antibiotic resistance genes.

Resistome analysis revealed the presence of 24 antimicrobial resistance genes (AMRGs) that are demonstrated in Table 1. *B. brasiliensis* sp. nov. carried 3 β-lactamase genes (*penP*, *bla*_TEM-16_, and *bla*_BKC-_1) genes; 4 aminoglycosides genes [*strA*, *strB*, *aac(6′)-Il*, *aac(6′)-Ib'*]; 2 genes that encode multidrug efflux pumps, which confer resistance to fluoroquinolones (*oqxBgb* and *adeF*); 3 genes that encode resistance-nodulation-cell division (RND)-based tripartite efflux pump (AcrA, AcrB, and TolC) capable of conferring resistance to a broad spectrum of antibiotics including β-lactams, tetracycline, novobiocin, and fluroquinolones; 2 genes associated to tetracycline and erythromycin resistance (*mexL* and *oprM*); 1 tetracyclines gene [*tet*(A)]; 1 glycopeptide gene (*van*Y); 1 trimethoprim gene (*dfr*A21); 1 sulfonamide gene (*sul1*); 1 cationic antimicrobial peptides such as daptomycin (*mprF*); and 1 amphenicol gene (*floR*).

Additionally, we found amino acid alterations in PhoP (Arg81Cis) and *qseC* (Ile283Leu) that mediate resistance to colistin antibiotics, as well as double amino acid substitution in GyrA (S83L and D87H) and single amino acid substitution in GyrB (Leu-466), which are associated with quinolone resistance (see Fig. S1 in the supplemental material).

### Virulence factors.

The distribution of genes associated with virulence factors is shown in Table 2. *Brevundimonas brasiliensis* sp. nov. displayed 18 genes that encode virulence-associated factors, including biofilm formation (*carB*, *ricA*, *msrAB*, *clpP*), adhesion (*cheB*, *bpsC*), invasion (*FliP*, *FliI*, *virB11*), lipopolysaccharide (LPS)/O-antigen synthesis (*gmd*), synthesis of siderophore (*dhbA*, *dhbE*), isocitrate lyase (*icl*), elongation factor Tu (*tufA*), 2-dehydro-3-deoxyphosphooctonate aldolase (*kdsA*), heat shock protein (*htpB*), and secretion (*gspG*, *xcpR*).

**TABLE 2:** Virulence determinants in *Brevundimonas brasiliensis* sp. nov.

Gene identifier	Putative gene	Encoding	Size (aa)	Species	Amino acid identity (%)
YP_170571	*carB*	Carbamoyl phosphate synthase large subunit	1,099	Francisella tularensis subsp. tularensis SCHU S4	58.99
NP_539653	*ricA*	Rab2 interacting conserved protein A	176	Brucella melitensis bv. 1 str. 16M	54.91
NP_273110	*msrAB* (*pilB*)	Trifunctional thioredoxin/methionine sulfoxide reductase A/B protein	147	Neisseria meningitidis MC58	60.99
NP_465991	*clpP*	ATP-dependent Clp protease proteolytic subunit	213	Listeria monocytogenes EGD-e	53.19
YP_001006775	*cheB*	Chemotaxis-specific methylesterase CheB	345	Yersinia enterocolitica subsp. enterocolitica 8081	50.86
AJI07989.1	*bpsC*	UTP-glucose-1-phosphate uridylyltransferase	297	Bacillus cereus G9241	51.58
YP_989394	*fliP*	Flagellar biosynthetic protein FliP	263	Bartonella bacilliformis KC583	50.41
YP_855907	*fliI*	Flagellum-specific ATP synthase	466	Aeromonas hydrophila subsp. hydrophila ATCC 7966	50.12
YP_034060	*virB11*	Type IV secretion system virB11 protein homolog	355	Bartonella henselae str. Houston-1	51.81
YP_034060	*virB11*	Type IV secretion system virB11 protein homolog	336	Bartonella henselae str. Houston-1	54.08
YP_001007257	*gmd*	GDP-mannose 4,6-dehydratase	325	Yersinia enterocolitica subsp. enterocolitica 8081	51.57
NP_832065	*dhbA*	2,3-dihydroxybenzoate-2,3-dehydrogenase, DhbA	256	Bacillus cereus ATCC 14579	52.76
NP_832067	*dhbE*	2,3-dihydroxybenzoate adenylase DhbE	256	Bacillus cereus ATCC 14579	63.99
YP_177728	*icl*	Isocitrate lyase Icl	425	Mycobacterium tuberculosis H37Rv	54.08
YP_169203.1	*tufA*	Elongation factor Tu	396	Francisella tularensis subsp. tularensis SCHU S4	78.28
NP_539767	*kdsA*	2-dehydro-3-deoxyphosphooctonate aldolase	300	Brucella melitensis bv. 1 str. 16M	66.79
YP_094724	*htpB*	Hsp60, 60K heat shock protein HtpB	548	Legionella pneumophila subsp. pneumophila str. Philadelphia 1	65.97
YP_404603	*gspG*	General secretion pathway protein G	172	Shigella dysenteriae Sd197	54.75
NP_251793	*xcpR*	General secretion pathway protein E	497	Pseudomonas aeruginosa PAO1	54.36

### Mobile genetic elements.

**FIG 5:**
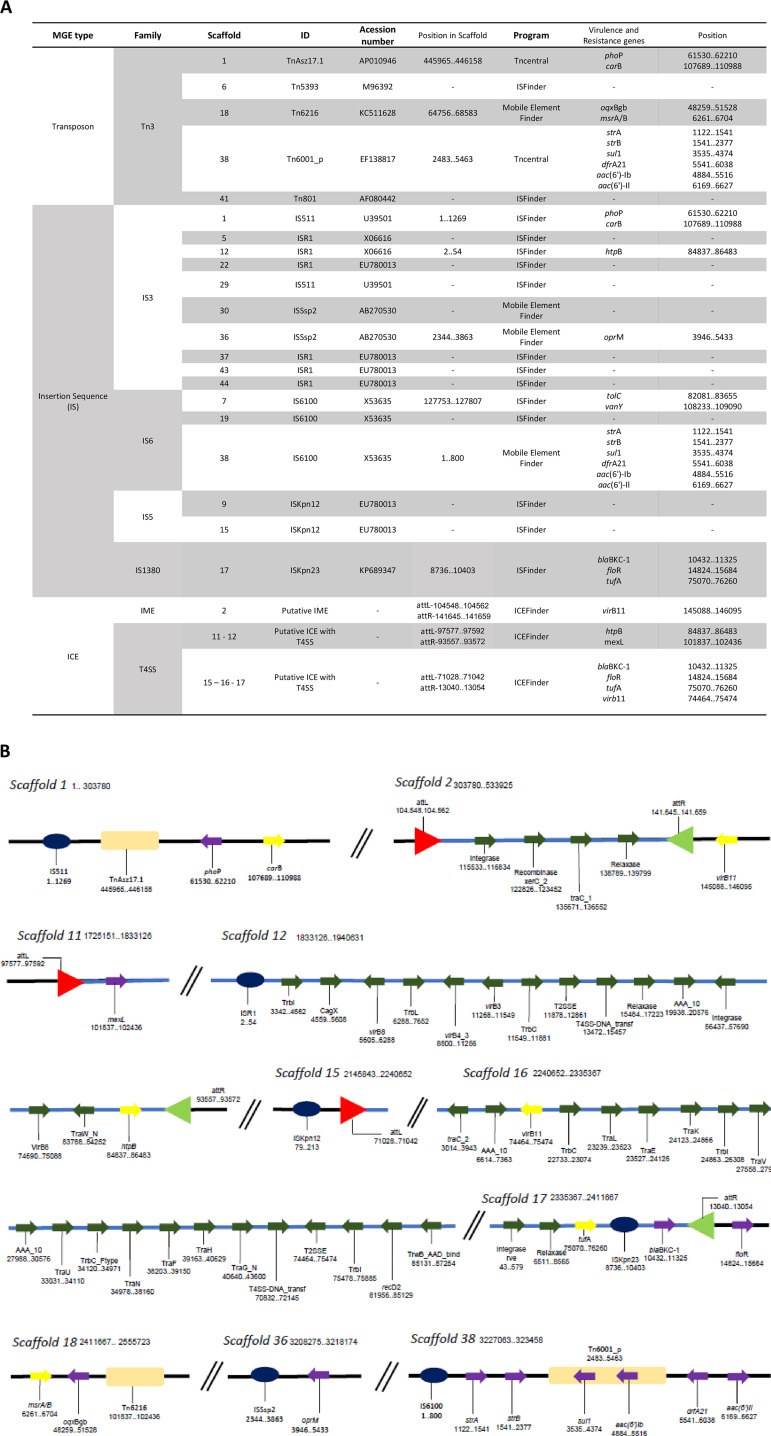
Mobile genetic elements (MGEs) in *Brevundimonas brasiliensis* sp. nov strain. (A) Genetic composition of the MGEs. (B) Schematic representation of MGEs identified in *B. brasiliensis* sp. nov. Black line represents the DNA strand, and the blue line represents the ICEs. The red and light green triangles are the attLs and attRs, respectively. The light orange rectangles are the transposons, and the dark blue circles are the insertion sequences. The genes are represented by arrows, the dark green arrows are the genes referring to conjugation of the ICEs, the yellow arrows are virulence genes, and the purple arrows are resistance genes.

The *B. brasiliensis* sp. nov. assembly was also examined for mobile genetic elements. The genome contained 2 copies of putative type IV secretion system (T4SS)-type integrative and conjugative elements (ICEs); 1 putative IME; 10 members (two copies of IS*511*, two copies of IS*Ssp2*, and six copies IS*R1*) of the IS*3* family; 3 members (three copies of IS*6100*) of the IS*6* family; 2 members (2 copies of IS*Kpn12*) of the IS*5* family, 1 member (IS*Kpn23*) of the IS*1380* family; and Tn*3*-type transposable elements (Tn*Asz17.1*, Tn*5393*, Tn*6216*, Tn*6001_p*) (Fig. 5A). Scaffold 1 contained a Tn*Asz17.1*, IS*511*, *phoP* resistance gene, and *carB* virulence gene. Scaffold 2 contained a *virB11* virulence gene, 1 IME encoding integrase, *xerC2* recombinase, and *traC1*, and it was bordered by a 15-bp direct repeat (attL, 5′-GGCGGTGATCGGCCC-3′; and attR, 5′-GGCGGTGATCGGCCC-3′) at both ends. In scaffold 11, we found a 16-bp direct repeat (attL, 5′-GGCGGCGGCGGCGGCG-3′) and a copy of a *mexL* gene. Scaffold 12 carried one copy of *htpB* virulence gene and elements of the “backbone” of integrative and conjugative elements (ICEs), such as type IV secretion systems (T4SSs), Trb(s), *virB*(s), insertions (IS*R1*), integrase, relaxase, and a 16-bp direct repeat attR (5′-GGCGGCGGCGGCGGCG-3′). Scaffold 15 harbored one copy of the IS*Kpn12* insertion element and a 15-bp direct repeat (attL, 5′-TGCTGGATCGCGCCG-3′). Scaffold 16 carried one copy of the *virB11* virulence gene and elements of the ICEs. Scaffold 17 contained integrase, relaxase, IS*Kpn23*, *bla*_BKC-1_ resistance gene, *tufA* virulence gene, a 15-bp direct repeat (attR, 5′-TGCTGGATCGCGCCG-3′), and one copy of *floR* resistance bordered the attR end. Tn*6216*, a resistance gene, and a virulence gene were present in scaffold 18. Scaffold 18 contained Tn*6216*, *oqxBgb* resistance, and *msrAB* virulence genes. Scaffold 36 carried only an IS*Ssp2* element and one copy of an *oprM* resistance gene. Scaffold 38 comprised the transposon-like element named Tn*6001_p*, the insertion sequence IS*6100*, and *strA*, *strB*, *aac(6’)-Ib*, *aac(6’)-Il*, *sul1*, *dfrA21* resistance genes (Fig. 5B). GIs, integrons, and prophages were not identified in the genome of *B. brasiliensis* sp. nov.

## DISCUSSION

The prevalence of certain MDR Gram-negative bacteria is increasing dramatically in patient care settings (25). Here, we reported a phenotypic and a systematic genomic characterization of a *Brevundimonas* clinical strain isolated from the cerebrospinal fluid of an infant admitted in the neonatal intensive care unit (NICU). Although it is considered a rare human pathogen, there has been an increase of infections caused by *Brevundimonas* spp. in recent years (26, 27), including in hospitalized children. Explanations for this include the following. Babies are more vulnerable to colonization and infection with pathogens due to an immature immune system. Novel molecular and phenotypic methods are providing more accurate and robust identification of these pathogens (28–30).

As *Brevundimonas* spp. are becoming known for their resistance properties to many different antibiotics (10, 31, 32), we analyzed the resistance profile to the antibiotics most commonly used to treat infections caused by Gram-negative bacteria. The studied *Brevundimonas* sp. was classified as MDR, presenting resistance to β-lactams, polymyxin, aminoglycosides, and fluoroquinolones. In contrast, we only observed susceptibility to tigecycline. Although the resistance mechanisms in the *Brevundimonas* genus remain poorly understood (10), it is known that the resistance profile can be highly varied. For instance, Brevundimonas vesicularis and Brevundimonas diminuta are the main species isolated from human infections (26). Studies have reported that both species may be resistant (17, 31, 32) or susceptible (9, 26, 33) to most antibiotics tested in this study.

Since species identification of the *Brevundimonas* isolate was not possible with the Vitek 2 system, WGS was performed on the *Brevundimonas* sp. for a more accurate identification and characterization of the isolate. The genome size and GC content were similar to most of the *Brevundimonas* spp. deposited in NCBI. The RAST and eggNOG analysis showed that most genes were related to cellular processes, which are essential to the bacteria (34). Notably, the genes related to the defense mechanisms present in eggNOG and disease in RAST analysis were associated with a multidrug resistance profile.

Preliminary phylogenetic analysis based on 16S rRNA gene sequences confirmed that our strain belongs to the genus *Brevundimonas.* It shared the highest similarity to *Brevundimonas olei* MJ15 (99.71%) followed by Brevundimonas naejangsanensis BIO TAS2-2 (99.37%). Although the 16S rRNA gene is widely used to differentiate strains at the genus level (35–37), it has poor discriminatory power at the species level since 16S rRNA genes are identical or highly homologous among different species (38). To aid bacterial identification, biochemical tests were performed, revealing that our strain is an oxidase positive and motile bacillus, unlike *Brevundimonas olei*. Multilocus sequence analysis (MLSA) based on several housekeeping genes has become a high-resolution technique to elucidate taxonomic relationship and phylogenetic analysis of closely related strains and subspecies (39, 40). The MLSA scheme based on five housekeeping genes (*atpD*, *recA*, *ileS*, *rpoD*, and *trpB*) showed that the *Brevundimonas* sp. isolate was clearly separated from Brevundimonas naejangsanensis FS1091 and Brevundimonas naejangsanensis DSM 23858, indicating a novel species within the *Brevundimonas* genus.

ANI or dDDH analysis has been most widely used as a gold standard for species delineation (24). Studies have reported that dDDH is considered necessary when strains share more than 97% 16S rRNA gene sequence similarity (41, 42), as it was observed for *Brevundimonas* sp., *Brevundimonas olei*, and Brevundimonas naejangsanensis BIO TAS2-2. To provide more accurate evidence to support that the *Brevundimonas* sp. strain is a novel species, ANI and dDDH analyses were performed with the *Brevundimonas* sp. and complete genomic and reference sequences from the genus *Brevundimonas* available in GenBank. Our data revealed that values for ANI (<95%) and dDDH (<70%) were lower than those generally accepted for species-level, showing that the isolate *Brevundimonas* sp. represents a novel species. To further validate our results, a phylogenetic tree inferred with genome BLAST distance phylogeny (GBDP) was constructed with the Type (Strain) Genome Server (TYGS), using the strain *Brevundimonas* sp. and reference sequences deposited in GenBank. The TYGS results also indicate that the strain *Brevundimonas* sp. is a novel species. Based on these findings, the name *Brevundimonas brasiliensis* sp. nov. was proposed.

To gain insights into similarity and distance within the genus *Brevundimonas*, we constructed two phylogenetic trees based on the set of core and accessory genomes (19). *Brevundimonas brasiliensis* sp. nov., *B. naejangsanensis* DSM 23858, *B. naejangsanensis* strain FS1091, and *B. naejangsanensis* B1 were grouped into the same clade in both trees. However, the phylogenetic trees presented a different topology. *Brevundimonas brasiliensis* sp. nov. showed evolutionary relatedness to the *B. naejangsanensis* DSM 23858 on the core gene tree, but they were no longer sisters on the accessory genome tree, suggesting that noncore genes were likely to make them diverged. KEGG analysis showed that most important pathways in core, accessory, and unique genes among four *Brevundimonas* strains are associated with “metabolism.” Among these genes, most were related to “amino acid metabolism,” “carbohydrate metabolism,” and “overview,” suggesting important roles in the maintenance of cellular function and survival. Important drug resistance genes were identified in unique gene clusters for human disease. *Brevundimonas brasiliensis* sp. nov. also harbored the highest number of singletons among the four strains, presenting specific genes associated with resistance and virulence genes. Singleton genes such as species-specific or strain-specific genes are those present in only one genome, which are usually acquired by horizontal gene transfer (43). All of these genomic features suggest a high versatility of *Brevundimonas* species in adapting to a wide range of environments, including health care environments.

We checked if the presence of AMRGs corresponded to phenotypic profiles and observed that the β-lactams in *Brevundimonas brasiliensis* sp. nov can be associated with *penP*, *bla*_TEM-16_, and *bla*_BKC-1_ genes. The *penP* gene encodes a narrow-spectrum β-lactamase that displays a more effective hydrolysis only of first—and second—generation penicillins and cephalosporins (44–46). The *bla*_TEM-116_ gene has been reported in a variety of clinical isolates (28, 47). Studies have related that TEM-116 β-lactamase can confer resistance to ceftazidime, cefotaxime, and aztreonam (48, 49). The *bla*_BKC-1_ gene encodes a Brazilian *Klebsiella* carbapenemase (BKC-1) that can confer resistance to penicillins, broad-spectrum cephalosporins, and aztreonam and decreased susceptibility to carbapenems (50). Interestingly, BKC-1 was described for the first time in Brazil in three Klebsiella pneumoniae strains (51) and more recently in a Citrobacter freundii strain (52), further showing that the *bla*_BKC-1_ gene is spreading to other pathogens.

The strain *B. brasiliensis* sp. nov also carried *strA*, *strB*, *aac(6’)-Ib*, *aac(6’)-Il* genes, which mediate resistance to aminoglycosides*. strA* and *strB*, which encode aminoglycoside-3″-phosphotransferase and aminoglycoside-6-phosphotransferase, form a tandem gene pair that confers resistance to many aminoglycoside antibiotics (53, 54). This gene pair can be found worldwide among diverse Gram-negative bacteria (55, 56). The *aac(6′)-Ib* gene is widely distributed among Gram-negative isolates, including Brevundimonas diminuta (31), and the main resistance mechanism to amikacin and other aminoglycosides is acetylation by the aminoglycoside 6′-*N*-acetyltransferase type Ib [*aac(6′)-Ib*] (57, 58).

Colistin resistance in Gram-negative bacteria can be attributed to mutation in PhoPQ, PmrAB, *qse*C, and plasmid-borne genes, such as *mcr* and its variants (59–61). Our strain displayed amino acid alterations at position 283 in *qseC* (Ile283Leu) and position 81 in *phoP* (Arg81Cis). Similar mutations have been reported by Pitt et al. (62) as conferring colistin resistance in K. pneumoniae.

Resistance to quinolones is frequently acquired by mutations in the quinolone resistance-determining regions (QRDRs) of the target genes, such as *gyrA*, *gyrB*, *parC*, and *parE* (28, 63). Our strain displayed a double amino acid substitution in GyrA, serine to leucine at codon 83, and aspartic acid to histidine at 87 (GyrA-S83L-D87H). Studies have reported that Ser83-Leu substitution in GyrA is usual, but an additional mutation in codon 87 is associated with higher levels of quinolone resistance than mutations at other codons within the QRDR (63, 64). Although the GyrB subunit is less commonly associated with quinolone resistance (65), *B. brasiliensis* sp. nov presented amino acid substitutions at position 466 in GyrB (Glu466-Leu). Similar findings have been reported in quinolone-resistant *B. diminuta* (32).

Antimicrobial resistance can also be acquired by altered expression of porins leading to decreased penetration of antibiotic within bacteria or increased efflux of antibiotics from the bacterial cell due to overexpression of efflux pump acting synergistically with the outer membrane mutation (66). The *oqxBgb* gene present in *Brevundimonas brasiliensis* sp. nov. can encode proteins that are part of multidrug efflux pumps responsible for fluoroquinolone resistance (67, 68) The *acrA*-like, *acrB*-like, and *tolC*-like genes found in *B. brasiliensis* sp. nov. encode a well-studied RND-based tripartite efflux pump (AcrAB-TolC) in Escherichia coli, which is able to export chloramphenicol, fluoroquinolone, tetracycline, rifampin, novobiocin, fusidic acid, nalidixic acid, and β-lactam antibiotics (69–71). *Brevundimonas brasiliensis* sp. nov. also carried *oprM* and *mexL* genes. OprM is the outer membrane component present in Burkholderia vietnamiensis and Pseudomonas aeruginosa (72, 73). This outer membrane protein is a component of MexAB-OprM, MexXY-OprM, MexJK-OprM, and MexVW-OprM efflux systems, and it mediates multidrug resistance in P. aeruginosa (74, 75). Although *mexAB*, *mexXY*, *mexJK*, and *mexVW* genes were not found in our strain, the *mexL* encoded a TetR family repressor (MexL) that is a negative regulator of MexJK expression that can be associated to tetracycline and erythromycin resistance (76–78).

Additionally, we found genes that can encode resistance toward macrolide tetracyclines *tet(A)* (79), glycopeptide (*vanY*) (80), trimethoprim (*dfrA21*) (81), florfenicol (*floR*) (82), sulfonamides (*sul1*) (83), and cationic antimicrobial peptides such as daptomycin (*mprF*) (84). Nonetheless, antimicrobial susceptibility testing was not performed since these antibiotics are not a common option for treating *Brevundimonas* spp. infections (10).

In *Brevundimonas* species, little is known about virulence factors contributing to pathogenicity. In our study, *B. brasiliensis* sp. nov. possessed an array of genes encoding virulence factors previously identified in many bacteria, and which are directly or indirectly related to biofilm formation (*carB* [85], *ricA* [86], *msrAB* [11], and *clpP* [87, 88]), adhesion (*cheB* [87] and *bpsC* [89]), invasion (*fliP* and *fliI* [90]; *virB11*, [91]), LPS/O-antigen synthesis (*gmd* [92]), synthesis of siderophore (*dhbA* and *dhbE* [93]), and secretion and transport (*gspG* [94] and *xcpR* [95]). Our strain also carried genes that encode isocitrate lyase (*icl*), elongation factor Tu (*tufA*), 2-dehydro-3-deoxyphosphooctonate aldolase (*kdsA*), and heat shock protein (*htpB*). Huang et al. (19) reported similar results regarding the occurrence of these virulence-associated genes in *Brevundimonas* spp.

Mobile genetic elements (MGEs) play an important role in the dissemination of antibiotic resistance and emergence of MDR pathogens worldwide (96). Still, the distribution of mobile genetic elements in the *Brevundimonas* genus remains scarce. In our study, whole-genome assemblies of *B. brasiliensis* sp. nov. presented several MGEs that can be associated with antibiotic resistance and/or virulence, including transposons, insertions, putative ICE with T4SS, and putative IME. Antibiotic gene cassettes [*strA*, *strB*, *aac(6’)-Ib*, *aac(6’)-Il*, *sul1*, *dfrA21*], IS*6100*, and Tn*6001* were located closely at scaffold 38. IS*6100* plays a role in *strA* and *strB* expression in Xanthomonas campestris pv. *vesicatoria* (97) and have been identified in many bacteria (98). Although the *bla*_VIM-3_ gene was not found in our isolate, studies have shown that Tn*6001* can contain a *bla*_VIM-3_-harboring integron In*450* and is associated to the dissemination of carbapenem-nonsusceptible Pseudomonas aeruginosa and extensively drug-resistant P. aeruginosa (99, 100). Bouallègue-Godet et al. (101) showed that *dfrA21*, which encodes resistance to trimethoprim, may be located in plasmids and inserted as a single resistance cassette in a class I integron of Salmonella enterica. The *aac(6’)-Ib* gene, responsible for most amikacin-resistant strains, is usually found in integrons, transposons, plasmids, and chromosomes of different bacterial species (102–104). *Brevundimonas brasiliensis* sp. nov. also presented IS*Kpn23* and putative ICE with T4SS harboring resistance genes (*bla*_BKC-1_ and *floR*) and virulence genes (*tufA* and/or *virB11*). Studies have shown that IS*Kpn23* plays an important role in expression of *bla*_BKC-1_ of K. pneumoniae (51, 105). The *floR* gene has been described for the small plasmid p1807 (106) of *Glaesserella parasuis* and on the multidrug resistance region of an incomplete Tn*4371*-like integrative and conjugative element (ICE) in the P. aeruginosa chromosome (107). The *virB11* virulence gene in our strain was associated with IME and putative ICE with T4SS. Campylobacter jejuni carries the *virB11* gene localized on the pVir plasmid that encodes various genes that are homologous to a type IV secretion system (91, 108). In our study, plasmid sequences were not detected using WGS, so it is uncertain whether many virulence and resistance genes are localized on plasmids. Furthermore, we could not correlate all the detected antibiotic resistance or virulence genes to MGEs due to the lack of literature.

In conclusion, we characterized a novel species of *Brevundimonas*, which is capable of infecting patients admitted to neonatal intensive care units. Since cases of *Brevundimonas* infection are being reported with increasing frequency, our report provides valuable information on this novel species that may be useful for surveillance, particularly in health care settings.

## MATERIALS AND METHODS

### Bacterial isolate.

The *Brevundimonas* sp. was recovered from the cerebrospinal fluid of an infant hospitalized at the Neonatal Intensive Care Unit (NICU) of the Hospital Geral de Palmas, Palmas, Tocantins, Brazil. This isolate was sent to the Central Laboratory of Public Health of Tocantins-Brazil (LACEN/TO/BR), a health care facility from the Brazilian Ministry of Health that receives samples of antimicrobial resistance for surveillance. The sample was sent for identification and antimicrobial susceptibility testing using the Vitek 2 system (bioMérieux, Marcy l’Etoile, France). However, species identification of *Brevundimonas* sp. was not possible using the Vitek system. Identification of the bacterial isolate at the genus and species level was further analyzed using whole-genome sequencing (WGS) by our research group. We also used 49 representative and complete sequences of *Brevundimonas* type strains in this study. Data is available in GenBank as of June 2022 (https://www.ncbi.nlm.nih.gov/genbank/) (see Table S1 in the supplemental material).

### Antimicrobial susceptibility.

The drug susceptibility of the *Brevundimonas* sp. was performed using the Vitek 2 system (bioMérieux, Inc., Hazelwood, MO, United States) following the Clinical and Laboratory Standards Institute guidelines (Clinical and Laboratory Standards Institute) (109). Phenotypic detection for the production of carbapenemases was carried out by modified Hodge test, synergy test, and the EDTA test under the CLSI guidelines (109) as described elsewhere (110–113). The MIC values of colistin and tigecycline were determined by the broth microdilution method, and results were interpreted based on the European Committee on Antimicrobial Susceptibility Testing (EUCAST, 2021; https://www.eucast.org/) criteria. The *Brevundimonas* sp. isolate was tested for susceptibility against 16 antibiotics as follows: amikacin, ampicillin, ampicillin/sulbactam, cefepime, cefoxitin, ceftazidime, ceftriaxone, cefuroxime axetil, ciprofloxacin, colistin, ertapenem, gentamicin, imipenem, meropenem, piperacillin-tazobactam, and tigecycline. Multidrug-resistant (MDR) *Brevundimonas* sp. isolate was defined by nonsusceptibility to at least one agent in three or more antibiotic categories (114).

### DNA isolation and library preparation for sequencing.

Total DNA extraction was performed using the Wizard Genomic DNA purification kit (Promega, Madison, WI, United States). The quantification of DNA was made using NanoVue Plus (GE Healthcare Life Sciences, Marlborough, MA, United States). The integrity of DNA was verified by electrophoresis analysis. Bacterial DNA concentration was also measured fluorometrically (Qubit 3.0, kit Qubit dsDNA broad-range assay kit; Life Technologies, Carlsbad, CA, United States). Samples were submitted to sequencing reaction using 1 ng of total DNA. Nextera XT DNA library prep kit (Illumina, San Diego, CA, United States) was used for library production. The libraries were amplified using a short cycle PCR program. In the first PCR step, the index 1 (i7) adapters and index 2 (i5) adapters were added for sequencing cluster generation. The purification of the library was performed using 0.6× Agencourt AMPure XP beads (Beckman Coulter). For checking the library quality and DNA fragment size, samples were analyzed by electrophoresis on 1.5% agarose gel. The libraries were quantified with a fluorometric method Qubit 3.0 using Qubit dsDNA broad-range assay kit (Life Technologies, Carlsbad, CA, United States) and normalized to 4 nM by standard dilution method. Libraries were pooled, denatured by addition of 0.2 N NaOH, and diluted to the final concentration of 1.8 pM. A PhiX control reaction was made in the final concentration of 1.5 pM. The run-length was a paired-end run of 75 cycles for each read (2 × 75), plus up to eight cycles each for two index reads.

### Genome assembly and annotation.

Data quality of raw reads was checked with FastQC v0.11.4 and trimmed to improve quality with Trim Galore v0.4.1. Genome assembly was performed with SPAdes 3.2 (115), and assembly statistics were accessed with QUAST (116). Plasmid detection and assembly attempts were performed by plasmidSPAdes (https://cab.spbu.ru/software/plasmid-spades/) (117). The circle map plot was obtained using the CGView server (118) (https://cgview.ca/). The assembled draft genome was annotated using the rapid prokaryotic genome annotation program Prokka (119) and the Rapid Annotation using Subsystem Technology (RAST) server (120) (https://rast.nmpdr.org/). The BUSCO program was used to evaluate the completeness of the assembled genome (121).

### COG eggNOG and GO annotation.

The COG (clusters of orthologous group) functional categories were annotated through online tool eggNOG-mapper (122) (http://eggnog-mapper.embl.de). Gene ontology (GO) was annotated through Blast2GO (123).

### 16S rRNA phylogeny and biochemical identification.

We identified a 16s rRNA gene sequence from our genome annotation. All curated 16S rRNA gene sequences from genus *Brevundimonas* were searched for in the GenBank database (see Table S2 in the supplemental material). The nucleotide sequences of 16s rRNA were aligned using multiple sequence alignment software (MAFFT) (124) (https://www.ebi.ac.uk/Tools/msa/mafft/). The construction of the maximum likelihood (ML) phylogenetic tree and the selection of the best assembly model were performed using the PhyML v3.0 program (125) and JModelTest (126), respectively.

The *Brevundimonas* sp. was subjected to biochemical tests using the Bactray I, II, III Systems according to the manufacturer's instructions (LaborClin, Paraná, Brazil). The results were compared with other *Brevundimonas* species reported in the literature.

### Multilocus sequence analysis.

Multilocus sequence analysis (MLSA) was conducted with five housekeeping genes, *atpD* (beta subunit of ATP synthase), *ileS* (isoleucina-tRNA ligase), *recA* (RecA protein), *rpoC* (DNA-directed RNA polymerase beta subunit), and *trpB* (beta chain of tryptophan synthase), which were retrieved from *Brevundimonas* reference species and the complete genome from the NCBI (National Center for Biotechnology Information) (https://www.ncbi.nlm.nih.gov/) (see Table S3 in the supplemental material). The genes were aligned and concatenated in the following order: *atpD*, *recA*, *ileS*, *rpoC*, and *trpB*. The phylogenetic tree was built with the PhyML v3.0 program (125) based on the best model chosen by JModelTest (126).

### Nucleotide identity and digital DNA-DNA hybridization.

Reference and complete genomes of *Brevundimonas* species were retrieved from the NCBI (https://www.ncbi.nlm.nih.gov/) (see Table S4 in the supplemental material). The average nucleotide identity (ANI) was calculated using the OrthoANI algorithm (127) (https://www.ezbiocloud.net/tools/ani). The digital DNA-DNA hybridization (dDDH) was calculated *in silico* by the Genome to Genome Distance Calculator (GGDC 3.0) using the BLAST method (128) (https://ggdc.dsmz.de/ggdc.php#). The NCBI online tool CIMminer (https://discover.nci.nih.gov/cimminer/oneMatrix.do) was used to build a heatmap with the orthoANI and dDDH results.

### TYGS analysis.

For phylogenetic inference of our strain *Brevundimonas* sp. the Type (Strain) Genome Server (TYGS) (129) (https://tygs.dsmz.de) was used against reference genomes of the genus *Brevundimonas* (see Table S5 in the supplemental material).

### Core and accessory genome comparison.

The complete and reference genomes of the genus *Brevundimonas* (see Table S6 in the supplemental material) were analyzed together with *Brevundimonas brasiliensis* sp. nov. using the Roary pipeline to infer the core and accessory genome trees (130).

### Genome analysis with OrthoVenn and KEGG.

For these analyses, we used the species closest to our strain according to the genomic core tree. Whole-genome comparison analysis of *Brevundimonas brasiliensis* sp. nov. against the selected genomes of *Brevundimonas* was performed using the OrthoVenn2 web server (https://orthovenn2.bioinfotoolkits.net) (131). Annotation of high-level functions and other high-throughput metabolism data was performed by Bacterial Pangenome Analysis Pipeline (BPGA) (132) against the Kyoto Encyclopedia Genomics and Genes Database (KEGG) (133). Thus, detailed identification of core genes, accessory genes, and unique genes was possible.

### Characterization of resistance and virulence factors.

The draft genome was screened for the presence of antimicrobial resistance (AMR) genes with the Rapid Annotation using Subsystem Technology server (RAST) (120) (https://rast.nmpdr.org/). BLAST was performed using two databases as follows: the comprehensive antibiotic resistance database (CARD; https://card.mcmaster.ca/) (134) and the antibiotic resistance gene ANNotation (ARG-ANNOT database) (135).

The following online tools were used for better annotation of resistance genes: CARDonline (https://card.mcmaster.ca/), ResFinder (https://www.genomicepidemiology.org/) (136), and BacAnt (http://bacant.net) (137). The AMRFinderPlus 3.10 (138) tool developed by NCBI was used with the BLAST pipeline. Annotation of resistance factors was also done by the Kyoto Encyclopedia Genomics and Genes Database (KEGG) (133), using the online tool BlastKOALA (http://www.kegg.jp/blastkoala/) for functional characterization of resistance genes.

Fluoroquinolone resistance genes were identified from Prokka annotation and manually aligned (139) to the sequences *gyrA* of E. coli (AAG57360.1), *gyrA* of Salmonella enterica (AAL21173.1), *gyrB* of E. coli (AAG58896.1), and *gyrB* of Salmonella enterica (AAL22694.1). Genes providing colistin resistance were identified by Prokka annotation, and mutations were identified manually.

Virulence factors of the strain were annotated with BLAST, using the Virulence Factor Database (140).

### Mobile genetic elements.

For identification of integrons and transposons, TnCentral (https://tncentral.ncc.unesp.br/) (141) was used. The ISfinder online server was used to identify insertion sequences in our genome (https://isfinder.biotoul.fr/blast.php) (142). To identify mobile elements and analyze their relationship with virulence and resistance factors, we used MGEfinder (https://www.genomicepidemiology.org/) from the Technical University of Denmark (DTU). Integrative and conjugative elements (ICE) were analyzed and identified by ICEfinder (143) (https://bioinfo-mml.sjtu.edu.cn/ICEfinder/ICEfinder.html). Phage analysis was performed using the PHAge search tool (PHAST) (http://phast.wishartlab.com) (144), and short palindromic repeats were studied with CRISPRfinder (https://bio.tools/crisprfinder) (145).

### Ethics statement.

In this work, we did not access the medical records of the patient. The *Brevundimonas* sp. and the anonymous archival data related to sample type were obtained from the Central Laboratory of Public Health of Tocantins (LACEN/TO, data’s owner). The studies involving human participants were reviewed and approved by the Committee of Ethics in Human Research of the Federal University of São Carlos (CEHRFUSC), and the need for informed consent for conducting this study was waived by the committee (no. 1.595.268). Patient consent was not required since the data presented in this study do not relate to any specific person or persons. Written informed consent from the participants or their legal guardian/next of kin was not required to participate in this study in accordance with the national legislation and institutional requirements. Permission to conduct the present study was obtained from the Health Department of the State of Tocantins (Secretaria da Saúde do Estado do Tocantins – SESAU) and LACEN/TO.

### Data availability.

The raw reads are available in the Sequence Read Archive under BioProject accession number PRJNA882454. This strain was deposited at the Bacteria Collection from Environment and Health (CBAS) of the Oswaldo Cruz Foundation (FIOCRUZ) (http://cbas.fiocruz.br/), under the (accession number CBAS 910).

## References

[1] Segers P, Vancanneyt M, Pot B, Torck U, Hoste B, Dewettinck D, Falsen E, Kersters K, de Vos P. 1994. Classification of Pseudomonas diminuta Leifson and Hugh 1954 and Pseudomonas vesicularis Busing, Doll, and Freytag 1953 in Brevundimonas gen. nov. as Brevundimonas diminuta comb. nov. and Brevundimonas vesicularis comb. nov., respectively. Int J Syst Bacteriol 44:499–510. doi:10.1099/00207713-44-3-499. 8068543

[2] Penna VTC, Martins SAM, Mazzola PG. 2002. Identification of bacteria in drinking and purified water during the monitoring of a typical water purification system. BMC Public Health 2:13. doi:10.1186/1471-2458-2-13. 12182763 PMC122092

[3] Tsubouchi T, Shimane Y, Usui K, Shimamura S, Mori K, Hiraki T, Tame A, Uematsu K, Maruyama T, Hatada Y. 2013. Brevundimonas abyssalis sp. nov., a dimorphic prosthecate bacterium isolated from deep-subsea floor sediment. Int J Syst Evol Microbiol 63:1987–1994. doi:10.1099/ijs.0.043364-0. 23041635

[4] Tsubouchi T, Koyama S, Mori K, Shimane Y, Usui K, Tokuda M, Tame A, Uematsu K, Maruyama T, Hatada Y. 2014. Brevundimonas denitrificans sp. nov., a denitrifying bacterium isolated from deep subseafloor sediment. Int J Syst Evol Microbiol 64:3709–3716. doi:10.1099/ijs.0.067199-0. 25106926

[5] Choi J-H, Kim M-S, Roh SW, Bae J-W. 2010. Brevundimonas basaltis sp. nov., isolated from black sand. Int J Syst Evol Microbiol 60:1488–1492. doi:10.1099/ijs.0.013557-0. 19671711

[6] Wang J, Zhang J, Ding K, Xin Y, Pang H. 2012. Brevundimonas viscosa sp. nov., isolated from saline soil. Int J Syst Evol Microbiol 62:2475–2479. doi:10.1099/ijs.0.035352-0. 22140155

[7] Kang S-J, Choi N-S, Choi JH, Lee J-S, Yoon J-H, Song JJ. 2009. Brevundimonas naejangsanensis sp. nov., a proteolytic bacterium isolated from soil, and reclassification of Mycoplana bullata into the genus Brevundimonas as Brevundimonas bullata comb. nov. Int J Syst Evol Microbiol 59:3155–3160. doi:10.1099/ijs.0.011700-0. 19643873

[8] Lee MR, Huang YT, Liao CH, Chuang TY, Lin CK, Lee SW, Lai CC, Yu CJ, Hsueh PR. 2011. Bacteremia caused by Brevundimonas species at a tertiary care hospital in Taiwan, 2000–2010. Eur J Clin Microbiol Infect Dis 30:1185–1191. doi:10.1007/s10096-011-1210-5. 21461849

[9] Chi C, Fung C, Wong W, Liu C. 2004. Brevundimonas bacteremia: two case reports and literature review. Scand J Infect Dis 36:59–61. doi:10.1080/00365540310018879. 15000562

[10] Ryan MP, Pembroke JT. 2018. Brevundimonas spp: emerging global opportunistic pathogens. Virulence 9:480–493. doi:10.1080/21505594.2017.1419116. 29484917 PMC5955483

[11] Singh S, Bhatia BD, Ansary A, Gambhir P, Anand MR, Parakh M, Agarwal S, Roy MP, Walsh K, John TJ, Pothapregada S. 2015. Brevundimonas septicemia: a rare infection with rare presentation. Indian Pediatr 52:901–907. doi:10.1007/s13312-015-0743-6. 26499022

[12] Donofrio RS, Bestervelt LL, Saha R, Bagley ST. 2010. Quantitative real-time PCR and fluorescence in situ hybridization approaches for enumerating Brevundimonas diminuta in drinking water. J Ind Microbiol Biotechnol 37:909–918. doi:10.1007/s10295-010-0738-1. 20495940

[13] Estrela AB, Abraham W-R. 2010. Brevundimonas vancanneytii sp. nov., isolated from blood of a patient with endocarditis. Int J Syst Evol Microbiol 60:2129–2134. doi:10.1099/ijs.0.015651-0. 19880635

[14] Yang M-L, Chen Y-H, Chen T-C, Lin W-R, Lin C-Y, Lu P-L. 2006. Case report: infective endocarditis caused by Brevundimonas vesicularis. BMC Infect Dis 6:179. doi:10.1186/1471-2334-6-179. 17194310 PMC1780062

[15] Mondello P, Ferrari L, Carnevale G. 2006. Nosocomial Brevundimonas vesicularis meningitis. Infez Med 14:235–237. 17380092

[16] Pandit RT. 2012. Brevundimonas diminuta keratitis. Eye Contact Lens Sci Clin Pract 38:63–65. doi:10.1097/ICL.0b013e31821c04f7. 21617535

[17] Gupta P, Appannanavar S, Kaur H, Gupta V, Mohan B, Taneja N. 2014. Hospital acquired urinary tract infection by multidrug-resistant Brevundimonas vesicularis. Indian J Pathol Microbiol 57:486–488. doi:10.4103/0377-4929.138789. 25118754

[18] Shobha KL, Ramachandra L, Gowrish S, Nagalakshmi N. 2013. Brevundimonas diminuta causing urinary tract infection. WebmedCentral 4:WMC004411.

[19] Huang Z, Yu K, Xiao Y, Wang Y, Xiao D, Wang D. 2022. Comparative genomic analysis reveals potential pathogenicity and slow-growth characteristics of genus Brevundimonas and description of Brevundimonas pishanensis sp. nov. Microbiol Spectr 10:e02468-21. doi:10.1128/spectrum.02468-21. PMC9045160 35416704

[20] Liu L, Feng Y, Wei L, Zong Z. 2021. Genome-based taxonomy of Brevundimonas with reporting Brevundimonas huaxiensis sp. nov. Microbiol Spectr 9:e0011121. doi:10.1128/Spectrum.00111-21. 34232096 PMC8552745

[21] Revez J, Espinosa L, Albiger B, Leitmeyer KC, Struelens MJ. 2017. Survey on the use of whole-genome sequencing for infectious diseases surveillance: rapid expansion of European national capacities, 2015–2016. Front Public Health 5:347. doi:10.3389/fpubh.2017.00347. 29326921 PMC5741818

[22] Jeong JH, Kweon OJ, Kim HR, Kim T-H, Ha S, Lee M-K. 2021. A novel species of the genus Arsenicicoccus isolated from human blood using whole-genome sequencing. Ann Lab Med 41:323–327. doi:10.3343/alm.2021.41.3.323. 33303718 PMC7748104

[23] Wambui J, Cernela N, Stevens MJA, Stephan R. 2021. Whole genome sequence-based identification of Clostridium estertheticum complex strains supports the need for taxonomic reclassification within the species Clostridium estertheticum. Front Microbiol 12:727022. doi:10.3389/fmicb.2021.727022. 34589074 PMC8473909

[24] Hu S, Li K, Zhang Y, Wang Y, Fu L, Xiao Y, Tang X, Gao J. 2022. New insights into the threshold values of multi-locus sequence analysis, average nucleotide identity and digital DNA–DNA hybridization in delineating Streptomyces species. Front Microbiol 13:910277. doi:10.3389/fmicb.2022.910277. 35711787 PMC9195134

[25] Flannery DD, Chiotos K, Gerber JS, Puopolo KM. 2022. Neonatal multidrug-resistant Gram-negative infection: epidemiology, mechanisms of resistance, and management. Pediatr Res 91:380–391. doi:10.1038/s41390-021-01745-7. 34599280 PMC8819496

[26] Lupande-Mwenebitu D, Tshiyongo RK, Lunguya-Metila O, Lavigne J-P, Rolain J-M, Diene SM. 2021. First isolation and clinical case of Brevundimonas diminuta in a newborn with low birth weight, in Democratic Republic of Congo: a case report. Medicina (B Aires) 57:1227. doi:10.3390/medicina57111227. PMC8617665 34833445

[27] Karadag N, Karagol BS, Kundak AA, Dursun A, Okumus N, Tanır G, Zencıroglu A. 2012. Spectrum of Brevundimonas vesicularis infections in neonatal period: a case series at a tertiary referral center. Infection 40:509–515. doi:10.1007/s15010-012-0274-1. 22711597

[28] Damas MSF, Ferreira RL, Campanini EB, Soares GG, Campos LC, Laprega PM, Soares da Costa A, Freire CDM, Pitondo-Silva A, Cerdeira LT, Cunha AD, Pranchevicius M-CDS. 2022. Whole genome sequencing of the multidrug-resistant Chryseobacterium indologenes isolated from a patient in Brazil. Front Med (Lausanne) 9:931379. doi:10.3389/fmed.2022.931379. 35966843 PMC9366087

[29] Viswanathan R, Singh A, Mukherjee R, Sardar S, Dasgupta S, Mukherjee S. 2010. Brevundimonas vesicularis: a new pathogen in newborn. J Pediatr Infect Dis 05:189–191.

[30] Pitout JDD, Church DL. 2004. Emerging Gram-negative enteric infections. Clin Lab Med 24:605–626. doi:10.1016/j.cll.2004.05.006. 15325058

[31] Almuzara MN, Barberis CM, Rodríguez CH, Famiglietti AMR, Ramirez MS, Vay CA. 2012. First report of an extensively drug-resistant VIM-2 metallo-β-lactamase-producing Brevundimonas diminuta clinical isolate. J Clin Microbiol 50:2830–2832. doi:10.1128/JCM.00924-12. 22692741 PMC3421493

[32] Han XY, Andrade RA. 2005. Brevundimonas diminuta infections and its resistance to fluoroquinolones. J Antimicrob Chemother 55:853–859. doi:10.1093/jac/dki139. 15883180

[33] Chandra A, Das A, Sen M, Sharma M. 2017. Brevundimonas diminuta infection in a case of nephrotic syndrome. Indian J Pathol Microbiol 60:279–281. doi:10.4103/IJPM.IJPM_679_15. 28631656

[34] Liang C-Y, Yang C-H, Lai C-H, Huang Y-H, Lin J-N. 2019. Genomic features, comparative genomic analysis, and antimicrobial susceptibility patterns of Chryseobacterium arthrosphaerae strain ED882-96 isolated in Taiwan Genes (Basel) 10:309. doi:10.3390/genes10040309. 31010035 PMC6523182

[35] Hayashi Sant’Anna F, Bach E, Porto RZ, Guella F, Hayashi Sant’Anna E, Passaglia LMP. 2019. Genomic metrics made easy: what to do and where to go in the new era of bacterial taxonomy. Crit Rev Microbiol 45:182–200. doi:10.1080/1040841X.2019.1569587. 31148498

[36] Weisburg WG, Barns SM, Pelletier DA, Lane DJ. 1991. 16S ribosomal DNA amplification for phylogenetic study. J Bacteriol 173:697–703. doi:10.1128/jb.173.2.697-703.1991. 1987160 PMC207061

[37] Clarridge JE. 2004. Impact of 16S rRNA gene sequence analysis for identification of bacteria on clinical microbiology and infectious diseases. Clin Microbiol Rev 17:840–862. doi:10.1128/CMR.17.4.840-862.2004. 15489351 PMC523561

[38] Janda JM, Abbott SL. 2007. 16S rRNA gene sequencing for bacterial identification in the diagnostic laboratory: pluses, perils, and pitfalls. J Clin Microbiol 45:2761–2764. doi:10.1128/JCM.01228-07. 17626177 PMC2045242

[39] Dai H, Lu B, Li Z, Huang Z, Cai H, Yu K, Wang D. 2020. Multilocus sequence analysis for the taxonomic updating and identification of the genus Proteus and reclassification of Proteus genospecies 5 O’Hara et al. 2000, Proteus cibarius Hyun et al. 2016 as later heterotypic synonyms of Proteus terrae Behrendt et al. 2015. BMC Microbiol 20:152. doi:10.1186/s12866-020-01844-1. 32522175 PMC7288399

[40] Liu Y, Lai Q, Shao Z. 2017. A multilocus sequence analysis scheme for phylogeny of Thioclava bacteria and proposal of two novel species. Front Microbiol 8:1321. doi:10.3389/fmicb.2017.01321. 28751885 PMC5508018

[41] Tindall BJ, Rosselló-Móra R, Busse H-J, Ludwig W, Kämpfer P. 2010. Notes on the characterization of prokaryote strains for taxonomic purposes. Int J Syst Evol Microbiol 60:249–266. doi:10.1099/ijs.0.016949-0. 19700448

[42] Gillis M, Vandamme P, Vos PD, Swings J, Kersters K. 2015. Polyphasic taxonomy, p 1–10. *In* Bergey’s manual of systematics of archaea and bacteria. Wiley, Hoboken, NJ.

[43] Costa SS, Guimarães LC, Silva A, Soares SC, Baraúna RA. 2020. First steps in the analysis of prokaryotic pan-genomes. Bioinform Biol Insights 14:117793222093806. doi:10.1177/1177932220938064. PMC7418249 32843837

[44] Pan X, He Y, Lei J, Huang X, Zhao Y. 2017. Crystallographic snapshots of class A β-lactamase catalysis reveal structural changes that facilitate β-lactam hydrolysis. J Biological Chemistry 292:4022–4033. doi:10.1074/jbc.M116.764340. PMC5354505 28100776

[45] Pan X, He Y, Chen T, Chan K-F, Zhao Y. 2017. Modified penicillin molecule with carbapenem-like stereochemistry specifically inhibits class C β-lactamases. Antimicrob Agents Chemother 61:e01288-17. doi:10.1128/AAC.01288-17. PMC5700298 28971874

[46] Wong W-T, Chan K-C, So P-K, Yap H-K, Chung W-H, Leung Y-C, Wong K-Y, Zhao Y. 2011. Increased structural flexibility at the active site of a fluorophore-conjugated β-lactamase distinctively impacts its binding toward diverse cephalosporin antibiotics. J Biol Chem 286:31771–31780. doi:10.1074/jbc.M110.198895. 21705325 PMC3173089

[47] Jacoby GA, Bush K. 2016. The curious case of TEM-116. Antimicrob Agents Chemother 60:7000. doi:10.1128/AAC.01777-16. 28045664 PMC5075131

[48] Jeong SH, Bae IK, Lee JH, Sohn SG, Kang GH, Jeon GJ, Kim YH, Jeong BC, Lee SH. 2004. Molecular characterization of extended-spectrum beta-lactamases produced by clinical isolates of Klebsiella pneumoniae and Escherichia coli from a Korean nationwide survey. J Clin Microbiol 42:2902–2906. doi:10.1128/JCM.42.7.2902-2906.2004. 15243036 PMC446292

[49] Lahlaoui H, Dahmen S, Moussa M, Omrane B. 2011. First detection of TEM-116 extended-spectrum β-lactamase in a Providencia stuartii isolate from a Tunisian hospital. Indian J Med Microbiol 29:258–261. doi:10.4103/0255-0857.83909. 21860106

[50] Bonnin RA, Jousset AB, Emeraud C, Oueslati S, Dortet L, Naas T. 2021. Genetic diversity, biochemical properties, and detection methods of minor carbapenemases in Enterobacterales. Front Med (Lausanne) 7:616490. doi:10.3389/fmed.2020.616490. 33553210 PMC7855592

[51] Nicoletti AG, Marcondes MFM, Martins WMBS, Almeida LGP, Nicolás MF, Vasconcelos ATR, Oliveira V, Gales AC. 2015. Characterization of BKC-1 class A carbapenemase from Klebsiella pneumoniae clinical isolates in Brazil. Antimicrob Agents Chemother 59:5159–5164. doi:10.1128/AAC.00158-15. 26055384 PMC4538461

[52] Martins WMBS, Seco BMS, Sampaio JLM, Sands K, Toleman MA, Gales AC. 2020. Detection of BKC-1 in Citrobacter freundii: a clue to mobilisation in an IncQ1 plasmid carrying blaBKC-1. Int J Antimicrob Agents 56:106042. doi:10.1016/j.ijantimicag.2020.106042. 32479892

[53] Lee YS, Kim GH, Koh YJ, Jung JS. 2021. Identification of strA-strB genes in streptomycin-resistant Pseudomonas syringae pv. actinidiae biovar 2 strains isolated in Korea. Plant Pathol J 37:489–493. doi:10.5423/PPJ.NT.05.2021.0078. 34847635 PMC8632614

[54] Sundin GW, Wang N. 2018. Antibiotic resistance in plant-pathogenic bacteria. Annu Rev Phytopathol 56:161–180. doi:10.1146/annurev-phyto-080417-045946. 29856934

[55] Tancos KA, Villani S, Kuehne S, Borejsza-Wysocka E, Breth D, Carol J, Aldwinckle HS, Cox KD. 2016. Prevalence of streptomycin-resistant Erwinia amylovora in New York apple orchards. Plant Dis 100:802–809. doi:10.1094/PDIS-09-15-0960-RE. 30688602

[56] Petrova MA, Gorlenko ZM, Soina VS, Mindlin SZ. 2008. Association of the strA-strB genes with plasmids and transposons in the present-day bacteria and in bacterial strains from permafrost. Genetika 44:1281–1286. 18846827

[57] Magallon J, Chiem K, Tran T, Ramirez MS, Jimenez V, Tolmasky ME. 2019. Restoration of susceptibility to amikacin by 8-hydroxyquinoline analogs complexed to zinc. PLoS One 14:e0217602. doi:10.1371/journal.pone.0217602. 31141575 PMC6541283

[58] Ramirez M, Tolmasky M. 2017. Amikacin: uses, resistance, and prospects for inhibition. Molecules 22:2267. doi:10.3390/molecules22122267. 29257114 PMC5889950

[59] Nirwan PK, Chatterjee N, Panwar R, Dudeja M, Jaggi N. 2021. Mutations in two component system (PhoPQ and PmrAB) in colistin resistant Klebsiella pneumoniae from North Indian tertiary care hospital. J Antibiot 74:450–457. doi:10.1038/s41429-021-00417-2. 33820943

[60] Breland EJ, Zhang EW, Bermudez T, Martinez CR, Hadjifrangiskou M. 2017. The histidine residue of QseC is required for canonical signaling between QseB and PmrB in uropathogenic Escherichia coli. J Bacteriol 199:e00060-17. doi:10.1128/JB.00060-17. 28396353 PMC5573081

[61] Pitt ME, Elliott AG, Cao MD, Ganesamoorthy D, Karaiskos I, Giamarellou H, Abboud CS, Blaskovich MAT, Cooper MA, Coin LJM. 2018. Multifactorial chromosomal variants regulate polymyxin resistance in extensively drug-resistant Klebsiella pneumoniae. Microb Genom 4:e000158. doi:10.1099/mgen.0.000158. 29431605 PMC5885010

[62] Pitt ME, Cao MD, Butler MS, Ramu S, Ganesamoorthy D, Blaskovich MAT, Coin LJM, Cooper MA. 2019. Octapeptin C4 and polymyxin resistance occur via distinct pathways in an epidemic XDR Klebsiella pneumoniae ST258 isolate. J Antimicrob Chemother 74:582–593. doi:10.1093/jac/dky458. 30445429 PMC6376851

[63] Hopkins KL, Davies RH, Threlfall EJ. 2005. Mechanisms of quinolone resistance in Escherichia coli and Salmonella: recent developments. Int J Antimicrob Agents 25:358–373. doi:10.1016/j.ijantimicag.2005.02.006. 15848289

[64] Minarini LAR, Darini ALC. 2012. Mutations in the quinolone resistance-determining regions of gyrA and parC in Enterobacteriaceae isolates from Brazil. Braz J Microbiol 43:1309–1314. 24031957 10.1590/S1517-838220120004000010 PMC3769005

[65] Momen G, Aainouss A, Lamaammal A, Chettioui F, Blaghen M, Messoudi M, Belghmi K, Mouslim J, el Mzibri M, el Messaoudi MD, Khyatti M, Chaoui I. 2021. Molecular characterization of mutations associated with resistance to second line drugs in Mycobacterium tuberculosis patients from Casablanca, Morocco. Rev Inst Med Trop Sao Paulo 63:e19. doi:10.1590/S1678-9946202163019. 33787739 PMC7997671

[66] Rushdy AA, Mabrouk MI, Abu-Sef FA-H, Kheiralla ZH, All SMA, Saleh NM. 2013. Contribution of different mechanisms to the resistance to fluoroquinolones in clinical isolates of Salmonella enterica. Brazilian J Infect Dis 17:431–437. doi:10.1016/j.bjid.2012.11.012. PMC9428056 23742803

[67] Salam LB. 2020. Unravelling the antibiotic and heavy metal resistome of a chronically polluted soil. 3 Biotech 10:238. doi:10.1007/s13205-020-02219-z. PMC7205953 32405442

[68] Coyne S, Rosenfeld N, Lambert T, Courvalin P, Périchon B. 2010. Overexpression of resistance-nodulation-cell division pump AdeFGH confers multidrug resistance in Acinetobacter baumannii. Antimicrob Agents Chemother 54:4389–4393. doi:10.1128/AAC.00155-10. 20696879 PMC2944555

[69] Piddock LV. 2006. Multidrug-resistance efflux pumps - not just for resistance. Nat Rev Microbiol 4:629–636. doi:10.1038/nrmicro1464. 16845433

[70] Fernandes P, Ferreira BS, Cabral JMS. 2003. Solvent tolerance in bacteria: role of efflux pumps and cross-resistance with antibiotics. Int J Antimicrob Agents 22:211–216. doi:10.1016/S0924-8579(03)00209-7. 13678823

[71] Okusu H, Ma D, Nikaido H. 1996. AcrAB efflux pump plays a major role in the antibiotic resistance phenotype of Escherichia coli multiple-antibiotic-resistance (Mar) mutants. J Bacteriol 178:306–308. doi:10.1128/jb.178.1.306-308.1996. 8550435 PMC177656

[72] Shinoy M, Dennehy R, Coleman L, Carberry S, Schaffer K, Callaghan M, Doyle S, McClean S. 2013. Immunoproteomic analysis of proteins expressed by two related pathogens, Burkholderia multivorans and Burkholderia cenocepacia, during human infection. PLoS One 8:e80796. doi:10.1371/journal.pone.0080796. 24260482 PMC3829912

[73] Wong KKY, Brinkman FSL, Benz RS, Hancock REW. 2001. Evaluation of a structural model of Pseudomonas aeruginosa outer membrane protein OprM, an efflux component involved in intrinsic antibiotic resistance. J Bacteriol 183:367–374. doi:10.1128/JB.183.1.367-374.2001. 11114937 PMC94886

[74] Scoffone VC, Trespidi G, Barbieri G, Irudal S, Perrin E, Buroni S. 2021. Role of RND efflux pumps in drug resistance of cystic fibrosis pathogens. Antibiotics 10:863. doi:10.3390/antibiotics10070863. 34356783 PMC8300704

[75] Alcalde-Rico M, Olivares-Pacheco J, Alvarez-Ortega C, Cámara M, Martínez JL. 2018. Role of the multidrug resistance efflux pump MexCD-OprJ in the Pseudomonas aeruginosa quorum sensing response. Front Microbiol 9:2752. doi:10.3389/fmicb.2018.02752. 30532741 PMC6266676

[76] Depardieu F, Podglajen I, Leclercq R, Collatz E, Courvalin P. 2007. Modes and modulations of antibiotic resistance gene expression. Clin Microbiol Rev 20:79–114. doi:10.1128/CMR.00015-06. 17223624 PMC1797629

[77] Chuanchuen R, Narasaki CT, Schweizer HP. 2002. The MexJK efflux pump of Pseudomonas aeruginosa requires OprM for antibiotic efflux but not for efflux of triclosan. J Bacteriol 184:5036–5044. doi:10.1128/JB.184.18.5036-5044.2002. 12193619 PMC135324

[78] Chuanchuen R, Gaynor JB, Karkhoff-Schweizer R, Schweizer HP. 2005. Molecular characterization of MexL, the transcriptional repressor of the mexJK multidrug efflux operon in Pseudomonas aeruginosa. Antimicrob Agents Chemother 49:1844–1851. doi:10.1128/AAC.49.5.1844-1851.2005. 15855505 PMC1087665

[79] Jahantigh M, Samadi K, Dizaji RE, Salari S. 2020. Antimicrobial resistance and prevalence of tetracycline resistance genes in Escherichia coli isolated from lesions of colibacillosis in broiler chickens in Sistan, Iran. BMC Vet Res 16:267. doi:10.1186/s12917-020-02488-z. 32746815 PMC7397602

[80] Stogios PJ, Savchenko A. 2020. Molecular mechanisms of vancomycin resistance. Protein Science 29:654–669. doi:10.1002/pro.3819. 31899563 PMC7020976

[81] Aworh MK, Kwaga JKP, Hendriksen RS, Okolocha EC, Thakur S. 2021. Genetic relatedness of multidrug resistant Escherichia coli isolated from humans, chickens and poultry environments. Antimicrob Resist Infect Control 10:58. doi:10.1186/s13756-021-00930-x. 33757589 PMC7988975

[82] Lu J, Zhang J, Xu L, Liu Y, Li P, Zhu T, Cheng C, Lu S, Xu T, Yi H, Li K, Zhou W, Li P, Ni L, Bao Q. 2018. Spread of the florfenicol resistance floR gene among clinical Klebsiella pneumoniae isolates in China. Antimicrob Resist Infect Control 7:127. doi:10.1186/s13756-018-0415-0. 30410748 PMC6211440

[83] Domínguez M, Miranda CD, Fuentes O, de la Fuente M, Godoy FA, Bello-Toledo H, González-Rocha G. 2019. Occurrence of transferable integrons and sul and dfr genes among sulfonamide-and/or trimethoprim-resistant bacteria isolated from Chilean salmonid farms. Front Microbiol 10: 748. doi:10.3389/fmicb.2019.00748. 31031727 PMC6474311

[84] Lin L-C, Chang S-C, Ge M-C, Liu T-P, Lu J-J. 2018. Novel single-nucleotide variations associated with vancomycin resistance in vancomycin-intermediate Staphylococcus aureus. IDR 11:113–123. doi:10.2147/IDR.S148335. PMC5783010 29403293

[85] Zhuo T, Rou W, Song X, Guo J, Fan X, Kamau GG, Zou H. 2015. Molecular study on the carAB operon reveals that carB gene is required for swimming and biofilm formation in Xanthomonas citri subsp. citri. BMC Microbiol 15:225. doi:10.1186/s12866-015-0555-9. 26494007 PMC4619228

[86] Adusei-Danso F, Khaja FT, DeSantis M, Jeffrey PD, Dubnau E, Demeler B, Neiditch MB, Dubnau D. 2019. Structure-function studies of the Bacillus subtilis Ric proteins identify the Fe-S cluster-ligating residues and their roles in development and RNA processing. mBio 10:e01841-19. doi:10.1128/mBio.01841-19. 31530674 PMC6751060

[87] Huang L, Wang L, Lin X, Su Y, Qin Y, Kong W, Zhao L, Xu X, Yan Q. 2017. mcp, aer, cheB, and cheV contribute to the regulation of Vibrio alginolyticus (ND-01) adhesion under gradients of environmental factors. Microbiologyopen 6:e00517. doi:10.1002/mbo3.517. 28744982 PMC5727358

[88] Wang C, Li M, Dong D, Wang J, Ren J, Otto M, Gao Q. 2007. Role of ClpP in biofilm formation and virulence of Staphylococcus epidermidis. Microbes Infect 9:1376–1383. doi:10.1016/j.micinf.2007.06.012. 17890122

[89] Diale MO, Kayitesi E, Serepa-Dlamini MH. 2021. Genome in silico and in vitro analysis of the probiotic properties of a bacterial endophyte, Bacillus paranthracis strain MHSD3. Front Genet 12:672149. doi:10.3389/fgene.2021.672149. 34858466 PMC8631869

[90] Cornelis GR. 2006. The type III secretion injectisome. Nat Rev Microbiol 4:811–825. doi:10.1038/nrmicro1526. 17041629

[91] Bacon DJ, Alm RA, Burr DH, Hu L, Kopecko DJ, Ewing CP, Trust TJ, Guerry P. 2000. Involvement of a plasmid in virulence of Campylobacter jejuni 81–176. Infect Immun 68:4384–4390. doi:10.1128/IAI.68.8.4384-4390.2000. 10899834 PMC98329

[92] Böhme K, Heroven AK, Lobedann S, Guo Y, Stolle A-S, Dersch P. 2021. The small protein YmoA controls the Csr system and adjusts expression of virulence-relevant traits of Yersinia pseudotuberculosis. Front Microbiol 12:706934. doi:10.3389/fmicb.2021.706934. 34413840 PMC8369931

[93] May JJ, Wendrich TM, Marahiel MA. 2001. The dhb operon of Bacillus subtilis encodes the biosynthetic template for the catecholic siderophore 2,3-dihydroxybenzoate-glycine-threonine trimeric ester bacillibactin. J Biol Chem 276:7209–7217. doi:10.1074/jbc.M009140200. 11112781

[94] Krall LJ, Klein S, Boutin S, Wu CC, Sähr A, Stanifer ML, Boulant S, Heeg K, Nurjadi D, Hildebrand D. 2021. Invasiveness of Escherichia coli is associated with an IncFII plasmid. Pathogens 10:1645. doi:10.3390/pathogens10121645. 34959600 PMC8707275

[95] Saeb AT, David SK, Al-Brahim H. 2015. Correction to: “in silico detection” of virulence gene homologues in the human pathogen Sphingomonas Spp. Evol Bioinform Online 11:1. doi:10.4137/EBO.S23536. PMC4295911 25633538

[96] Nicolas E, Oger CA, Nguyen N, Lambin M, Draime A, Leterme SC, Chandler M, Hallet BFJ. 2017. Unlocking Tn3-family transposase activity in vitro unveils an asymetric pathway for transposome assembly. Proc Natl Acad Sci USA 114:E669–E678. doi:10.1073/pnas.1611701114. 28096365 PMC5293067

[97] Sundin GW, Bender CL. 1995. Expression of the strA-strB streptomycin resistance genes in Pseudomonas syringae and Xanthomonas campestris and characterization of IS6100 in X. campestris. Appl Environ Microbiol 61:2891–2897. doi:10.1128/aem.61.8.2891-2897.1995. 7487022 PMC167566

[98] Varani A, He S, Siguier P, Ross K, Chandler M. 2021. The IS6 family, a clinically important group of insertion sequences including IS26. Mob DNA 12:11. doi:10.1186/s13100-021-00239-x. 33757578 PMC7986276

[99] Tseng S-P, Tsai J-C, Teng L-J, Hsueh P-R. 2009. Dissemination of transposon Tn6001 in carbapenem-non-susceptible and extensively drug-resistant Pseudomonas aeruginosa in Taiwan. J Antimicrob Chemother 64:1170–1174. doi:10.1093/jac/dkp341. 19773253 PMC2775663

[100] Tseng S-P, Hsueh P-R, Tsai J-C, Teng L-J. 2007. Tn 6001, a transposon-like element Containing the blaVIM-3-harboring Integron In450. Antimicrob Agents Chemother 51:4187–4190. doi:10.1128/AAC.00542-07. 17846142 PMC2151417

[101] Bouallègue-Godet O, Ben Salem Y, Fabre L, Demartin M, Grimont PAD, Mzoughi R, Weill F-X. 2005. Nosocomial outbreak caused by Salmonella enterica serotype Livingstone producing CTX-M-27 extended-spectrum β-lactamase in a neonatal unit in Sousse, Tunisia. J Clin Microbiol 43:1037–1044. doi:10.1128/JCM.43.3.1037-1044.2005. 15750057 PMC1081247

[102] Ramirez MS, Nikolaidis N, Tolmasky ME. 2013. Rise and dissemination of aminoglycoside resistance: the aac(6′)-Ib paradigm. Front Microbiol 4:121. doi:10.3389/fmicb.2013.00121. 23730301 PMC3656343

[103] Ramirez MS, Tolmasky ME. 2010. Aminoglycoside modifying enzymes. Drug Resist Update 13:151–171. doi:10.1016/j.drup.2010.08.003. PMC2992599 20833577

[104] Reeves CM, Magallon J, Rocha K, Tran T, Phan K, Vu P, Yi Y, Oakley-Havens CL, Cedano J, Jimenez V, Ramirez MS, Tolmasky ME. 2021. Aminoglycoside 6′-N-acetyltransferase Type Ib [AAC(6′)-Ib]-mediated aminoglycoside resistance: phenotypic conversion to susceptibility by silver ions. Antibiotics 10:29. doi:10.3390/antibiotics10010029. PMC7824292 33396404

[105] Martins WMBS, Nascimento EA, Cayô R, Gales AC. 2022. Role of IS Kpn23 in blaBKC-1 expression and mobilization. Antimicrob Agents Chemother 66:e00875-21. doi:10.1128/aac.00875-21. 35311517 PMC9017360

[106] Sun H, Zhang J, Miao Q, Zhai Y, Pan Y, Yuan L, Yan F, Wu H, Hu G. 2022. Genomic insight into the integrative conjugative elements from ICEHpa1 family. Front Vet Sci 9:986824. doi:10.3389/fvets.2022.986824. 36061114 PMC9437646

[107] Qian C, Liu H, Cao J, Ji Y, Lu W, Lu J, Li A, Zhu X, Shen K, Xu H, Chen Q, Zhou W, Lu H, Lin H, Zhang X, Li Q, Lin X, Li K, Xu T, Zhu M, Bao Q, Zhang H. 2021. Identification of floR variants associated with a novel Tn4371-like integrative and conjugative element in clinical Pseudomonas aeruginosa isolates. Front Cell Infect Microbiol 11:685068. doi:10.3389/fcimb.2021.685068. 34235095 PMC8256890

[108] Sierra-Arguello YM, Perdoncini G, Rodrigues LB, Ruschel dos Santos L, Apellanis Borges K, Quedi Furian T, Pippi Salle CT, de Souza Moraes HL, Pereira Gomes MJ, Pinheiro do Nascimento V. 2021. Identification of pathogenic genes in Campylobacter jejuni isolated from broiler carcasses and broiler slaughterhouses. Sci Rep 11:4588. doi:10.1038/s41598-021-84149-1. 33633256 PMC7907142

[109] Clinical and Laboratory Standards Institute. 2021. Performance standards for antimicrobial susceptibility testing—32nd ed. Clinical and Laboratory Standards Institute, Wayne, PA.

[110] Miriagou V, Cornaglia G, Edelstein M, Galani I, Giske CG, Gniadkowski M, Malamou-Lada E, Martinez-Martinez L, Navarro F, Nordmann P, Peixe L, Pournaras S, Rossolini GM, Tsakris A, Vatopoulos A, Cantón R. 2010. Acquired carbapenemases in Gram-negative bacterial pathogens: detection and surveillance issues. Clin Microbiol Infect 16:112–122. doi:10.1111/j.1469-0691.2009.03116.x. 20085605

[111] Nordmann P, Naas T, Poirel L. 2011. Global spread of carbapenemase-producing Enterobacteriaceae. Emerg Infect Dis 17:1791–1798. doi:10.3201/eid1710.110655. 22000347 PMC3310682

[112] Ferreira RL, Rezende GS, Damas MSF, Oliveira-Silva M, Pitondo-Silva A, Brito MCA, Leonardecz E, de Góes FR, Campanini EB, Malavazi I, da Cunha AF, Pranchevicius M-CDS. 2020. Characterization of KPC-producing Serratia marcescens in an intensive care unit of a Brazilian tertiary hospital. Front Microbiol 11:956. doi:10.3389/fmicb.2020.00956. 32670210 PMC7326048

[113] Ferreira RL, da Silva BCM, Rezende GS, Nakamura-Silva R, Pitondo-Silva A, Campanini EB, Brito MCA, da Silva EML, Freire CDM, Cunha AD, Pranchevicius M-CDS. 2019. High prevalence of multidrug-resistant Klebsiella pneumoniae harboring several virulence and β-lactamase encoding genes in a Brazilian intensive care unit. Front Microbiol 9:3198. doi:10.3389/fmicb.2018.03198. 30723463 PMC6349766

[114] Magiorakos A-P, Srinivasan A, Carey RB, Carmeli Y, Falagas ME, Giske CG, Harbarth S, Hindler JF, Kahlmeter G, Olsson-Liljequist B, Paterson DL, Rice LB, Stelling J, Struelens MJ, Vatopoulos A, Weber JT, Monnet DL. 2012. Multidrug-resistant, extensively drug-resistant and pandrug-resistant bacteria: an international expert proposal for interim standard definitions for acquired resistance. Clin Microbiol Infect 18:268–281. doi:10.1111/j.1469-0691.2011.03570.x. 21793988

[115] Bankevich A, Nurk S, Antipov D, Gurevich AA, Dvorkin M, Kulikov AS, Lesin VM, Nikolenko SI, Pham S, Prjibelski AD, Pyshkin AV, Sirotkin AV, Vyahhi N, Tesler G, Alekseyev MA, Pevzner PA. 2012. SPAdes: a new genome assembly algorithm and its applications to single-cell sequencing. J Comput Biol 19:455–477. doi:10.1089/cmb.2012.0021. 22506599 PMC3342519

[116] Gurevich A, Saveliev V, Vyahhi N, Tesler G. 2013. QUAST: quality assessment tool for genome assemblies. Bioinformatics 29:1072–1075. doi:10.1093/bioinformatics/btt086. 23422339 PMC3624806

[117] Antipov D, Hartwick N, Shen M, Raiko M, Lapidus A, Pevzner PA. 2016. plasmidSPAdes: assembling plasmids from whole genome sequencing data. Bioinformatics 32:3380–3387. doi:10.1093/bioinformatics/btw493. 27466620

[118] Grant JR, Stothard P. 2008. The CGView server: a comparative genomics tool for circular genomes. Nucleic Acids Res 36:W181–W184. doi:10.1093/nar/gkn179. 18411202 PMC2447734

[119] Seemann T. 2014. Prokka: rapid prokaryotic genome annotation. Bioinformatics 30:2068–2069. doi:10.1093/bioinformatics/btu153. 24642063

[120] Aziz RK, Bartels D, Best AA, DeJongh M, Disz T, Edwards RA, Formsma K, Gerdes S, Glass EM, Kubal M, Meyer F, Olsen GJ, Olson R, Osterman AL, Overbeek RA, McNeil LK, Paarmann D, Paczian T, Parrello B, Pusch GD, Reich C, Stevens R, Vassieva O, Vonstein V, Wilke A, Zagnitko O. 2008. The RAST server: rapid annotations using subsystems technology. BMC Genomics 9:75. doi:10.1186/1471-2164-9-75. 18261238 PMC2265698

[121] Simão FA, Waterhouse RM, Ioannidis P, Kriventseva EV, Zdobnov EM. 2015. BUSCO: assessing genome assembly and annotation completeness with single-copy orthologs. Bioinformatics 31:3210–3212. doi:10.1093/bioinformatics/btv351. 26059717

[122] Cantalapiedra CP, Hernández-Plaza A, Letunic I, Bork P, Huerta-Cepas J. 2021. eggNOG-mapper v2: functional annotation, orthology assignments, and domain prediction at the metagenomic scale. Mol Biol Evol 38:5825–5829. doi:10.1093/molbev/msab293. 34597405 PMC8662613

[123] Conesa A, Götz S. 2008. Blast2GO: a comprehensive suite for functional analysis in plant genomics. Int J Plant Genomics 2008:1–12. doi:10.1155/2008/619832. PMC2375974 18483572

[124] Katoh K, Standley DM. 2013. MAFFT multiple sequence alignment software version 7: improvements in performance and usability. Mol Biol Evol 30:772–780. doi:10.1093/molbev/mst010. 23329690 PMC3603318

[125] Guindon S, Delsuc F, Dufayard J-F, Gascuel O. 2009. Estimating maximum likelihood phylogenies with PhyML. Methods Mol Biol 537:113–137. doi:10.1007/978-1-59745-251-9_6. 19378142

[126] Posada D. 2008. jModelTest: phylogenetic model averaging. Mol Biol Evol 25:1253–1256. doi:10.1093/molbev/msn083. 18397919

[127] Yoon S-H, Ha S-M, Kwon S, Lim J, Kim Y, Seo H, Chun J. 2017. Introducing EzBioCloud: a taxonomically united database of 16S rRNA gene sequences and whole-genome assemblies. Int J Syst Evol Microbiol 67:1613–1617. doi:10.1099/ijsem.0.001755. 28005526 PMC5563544

[128] Meier-Kolthoff JP, Auch AF, Klenk H-P, Göker M. 2013. Genome sequence-based species delimitation with confidence intervals and improved distance functions. BMC Bioinformatics 14:60. doi:10.1186/1471-2105-14-60. 23432962 PMC3665452

[129] Meier-Kolthoff JP, Carbasse JS, Peinado-Olarte RL, Göker M. 2022. TYGS and LPSN: a database tandem for fast and reliable genome-based classification and nomenclature of prokaryotes. Nucleic Acids Res 50:D801–D807. doi:10.1093/nar/gkab902. 34634793 PMC8728197

[130] Page AJ, Cummins CA, Hunt M, Wong VK, Reuter S, Holden MTG, Fookes M, Falush D, Keane JA, Parkhill J. 2015. Roary: rapid large-scale prokaryote pan genome analysis. Bioinformatics 31:3691–3693. doi:10.1093/bioinformatics/btv421. 26198102 PMC4817141

[131] Xu L, Dong Z, Fang L, Luo Y, Wei Z, Guo H, Zhang G, Gu YQ, Coleman-Derr D, Xia Q, Wang Y. 2019. OrthoVenn2: a web server for whole-genome comparison and annotation of orthologous clusters across multiple species. Nucleic Acids Res 47:W52–W58. doi:10.1093/nar/gkz333. 31053848 PMC6602458

[132] Chaudhari NM, Gupta VK, Dutta C. 2016. BPGA- an ultra-fast pan-genome analysis pipeline. Sci Rep 6:24373. doi:10.1038/srep24373. 27071527 PMC4829868

[133] Kanehisa M. 2000. KEGG: Kyoto Encyclopedia of Genes and Genomes. Nucleic Acids Res 28:27–30. doi:10.1093/nar/28.1.27. 10592173 PMC102409

[134] Jia B, Raphenya AR, Alcock B, Waglechner N, Guo P, Tsang KK, Lago BA, Dave BM, Pereira S, Sharma AN, Doshi S, Courtot M, Lo R, Williams LE, Frye JG, Elsayegh T, Sardar D, Westman EL, Pawlowski AC, Johnson TA, Brinkman FSL, Wright GD, McArthur AG. 2017. CARD 2017: expansion and model-centric curation of the comprehensive antibiotic resistance database. Nucleic Acids Res 45:D566–D573. doi:10.1093/nar/gkw1004. 27789705 PMC5210516

[135] Gupta SK, Padmanabhan BR, Diene SM, Lopez-Rojas R, Kempf M, Landraud L, Rolain J-M. 2014. ARG-ANNOT, a new bioinformatic tool to discover antibiotic resistance genes in bacterial genomes. Antimicrob Agents Chemother 58:212–220. doi:10.1128/AAC.01310-13. 24145532 PMC3910750

[136] Bortolaia V, Kaas RS, Ruppe E, Roberts MC, Schwarz S, Cattoir V, Philippon A, Allesoe RL, Rebelo AR, Florensa AF, Fagelhauer L, Chakraborty T, Neumann B, Werner G, Bender JK, Stingl K, Nguyen M, Coppens J, Xavier BB, Malhotra-Kumar S, Westh H, Pinholt M, Anjum MF, Duggett NA, Kempf I, Nykäsenoja S, Olkkola S, Wieczorek K, Amaro A, Clemente L, Mossong J, Losch S, Ragimbeau C, Lund O, Aarestrup FM. 2020. ResFinder 4.0 for predictions of phenotypes from genotypes. J Antimicrob Chemother 75:3491–3500. doi:10.1093/jac/dkaa345. 32780112 PMC7662176

[137] Hua X, Liang Q, Deng M, He J, Wang M, Hong W, Wu J, Lu B, Leptihn S, Yu Y, Chen H. 2021. BacAnt: a combination annotation server for bacterial DNA sequences to identify antibiotic resistance genes, integrons, and transposable elements. Front Microbiol 12:649969. doi:10.3389/fmicb.2021.649969. 34367079 PMC8343408

[138] Feldgarden M, Brover V, Gonzalez-Escalona N, Frye JG, Haendiges J, Haft DH, Hoffmann M, Pettengill JB, Prasad AB, Tillman GE, Tyson GH, Klimke W. 2021. AMRFinderPlus and the Reference Gene Catalog facilitate examination of the genomic links among antimicrobial resistance, stress response, and virulence. Sci Rep 11:12728. doi:10.1038/s41598-021-91456-0. 34135355 PMC8208984

[139] Giles JA, Falconio J, Yuenger JD, Zenilman JM, Dan M, Bash MC. 2004. Quinolone resistance–determining region mutations and por type of Neisseria gonorrhoeae isolates: resistance surveillance and typing by molecular methodologies. J Infect Dis 189:2085–2093. doi:10.1086/386312. 15143477

[140] Chen L, Zheng D, Liu B, Yang J, Jin Q. 2016. VFDB 2016: hierarchical and refined dataset for big data analysis—10 years on. Nucleic Acids Res 44:D694–D697. doi:10.1093/nar/gkv1239. 26578559 PMC4702877

[141] Ross K, Varani AM, Snesrud E, Huang H, Alvarenga DO, Zhang J, Wu C, McGann P, Chandler M. 2021. TnCentral: a prokaryotic transposable element database and web portal for transposon analysis. mBio 12:e02060-21. doi:10.1128/mBio.02060-21. 34517763 PMC8546635

[142] Siguier P, Perochon J, Lestrade L, Mahillon J, Chandler M. 2006. ISfinder: the reference centre for bacterial insertion sequences. Nucleic Acids Res 34:D32–D36. doi:10.1093/nar/gkj014. 16381877 PMC1347377

[143] Bi D, Xu Z, Harrison EM, Tai C, Wei Y, He X, Jia S, Deng Z, Rajakumar K, Ou H-Y. 2012. ICEberg: a web-based resource for integrative and conjugative elements found in bacteria. Nucleic Acids Res 40:D621–D626. doi:10.1093/nar/gkr846. 22009673 PMC3244999

[144] Zhou Y, Liang Y, Lynch KH, Dennis JJ, Wishart DS. 2011. PHAST: a fast phage search tool. Nucleic Acids Res 39:W347–W352. doi:10.1093/nar/gkr485. 21672955 PMC3125810

[145] Grissa I, Vergnaud G, Pourcel C. 2007. CRISPRFinder: a web tool to identify clustered regularly interspaced short palindromic repeats. Nucleic Acids Res 35:W52–W57. doi:10.1093/nar/gkm360. 17537822 PMC1933234

[146] Lee M, Srinivasan S, Kim MK. 2010. New taxa in Alphaproteobacteria: Brevundimonas olei sp. nov., an esterase-producing bacterium. J Microbiol 48:616–622. doi:10.1007/s12275-010-9367-7. 21046339

[147] Lefort V, Desper R, Gascuel O. 2015. FastME 2.0: a comprehensive, accurate, and fast distance-based phylogeny inference program. Mol Biol Evol 32:2798–2800. doi:10.1093/molbev/msv150. 26130081 PMC4576710

[148] Farris JS. 1972. Estimating phylogenetic trees from distance matrices. Am Nat 106:645–668. doi:10.1086/282802.

