# Supplementary material for: Brevundimonas brasiliensis sp. nov.: a New Multidrug-Resistant Species Isolated from a Patient in Brazil
Source: Microbiol Spectr. 2023 Apr 17;11(3):e04415-22. doi: 10.1128/spectrum.04415-22 (PMC10269605; doi:10.1128/spectrum.04415-22)
Supplement: Supplemental file 1 — Fig. 1 and Tables S1 to S6. Download spectrum.04415-22-s0001.pdf, PDF file, 0.4 MB [file spectrum.04415-22-s0001.pdf]

Supplementary

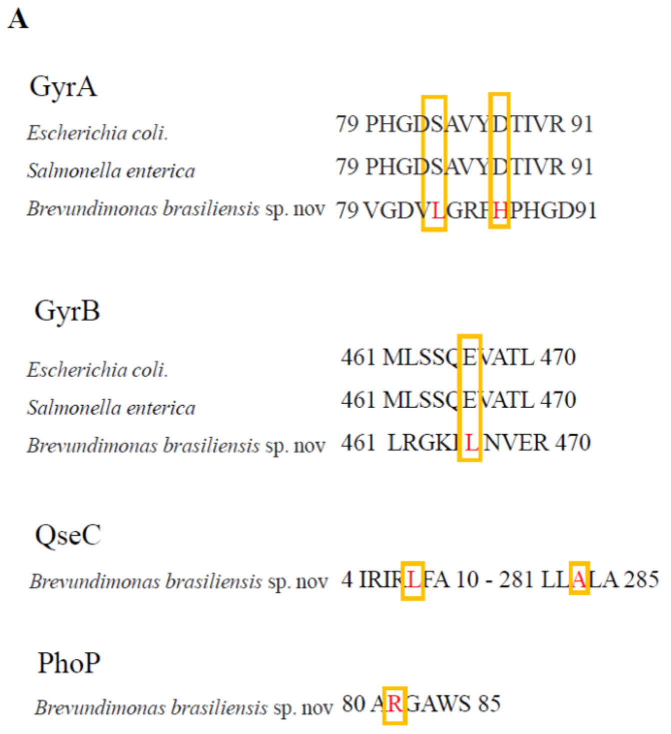

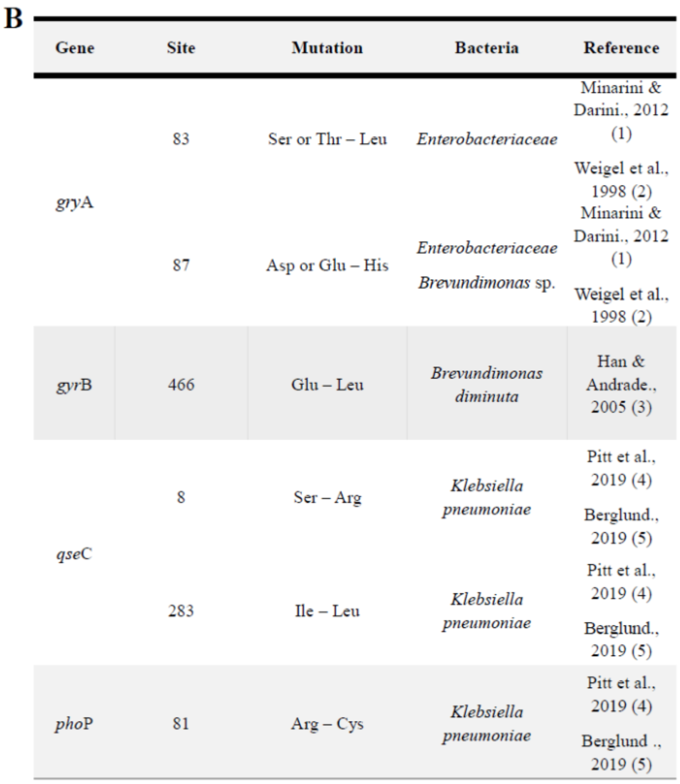
B

Gene	Site	Mutation	Bacteria	Reference
gryA	83	Ser or Thr – Leu	*Enterobacteriaceae*	Minarini & Darini., 2012 (1) Weigel et al., 1998 (2)
	87	Asp or Glu – His	*Enterobacteriaceae* *Brevundimonas* sp.	Minarini & Darini., 2012 (1) Weigel et al., 1998 (2)
gyrB	466	Glu – Leu	*Brevundimonas diminuta*	Han & Andrade., 2005 (3)
qseC	8	Ser – Arg	*Klebsiella pneumoniae*	Pitt et al., 2019 (4) Berglund., 2019 (5)
	283	Ile – Leu	*Klebsiella pneumoniae*	Pitt et al., 2019 (4) Berglund., 2019 (5)
phoP	81	Arg – Cys	*Klebsiella pneumoniae*	Pitt et al., 2019 (4) Berglund ., 2019 (5)

Figure S1. (A) Amino acid sequence alignment for the GyrA QRDR (Ser83Leu) of *Brevundimonas brasiliensis sp.nov*; GyrB QRDR (Glu466Leu) of *Brevundimonas brasiliensis sp.nov* ; and mutations in QseC (Ser8Arg) of *Brevundimonas brasiliensis sp.nov* and QseC (Ile283Leu) *Brevundimonas brasiliensis sp.nov* ; PhoP (Arg81Cys) *Brevundimonas brasiliensis sp.nov*. (B) Known mutations in genes conferring resistance to quinolones (GyrA and GyrB QDDR) and colistin (*qse*C and *pho*P).

Table S1. Reference and complete genomes used in this manuscript, including accession number.

**Species**	**Accession numbers**	**Genome**
Brevundimonas abyssalis TAR-001	GCF_000466985.1	Reference
Brevundimonas alba DSM 4736	GCF_011927945.1	Reference
Brevundimonas aurantiaca FXH-172	GCF_020782355.1	Reference
Brevundimonas albigilva T7	GCF_023573565.1	Complete
Brevundimonas aveniformis DSM 17977	GCF_000428765.1	Reference
Brevundimonas bacteroides DSM 4726	GCF_000701445.1	Reference
Brevundimonas basaltis DSM 25335	GCF_014202075.1	Reference
Brevundimonas bullata HAMBI_262	GCF_003350205.1	Reference
Brevundimonas diminuta ATCC(B) 19146	GCF_004102925.1	Complete
Brevundimonas diminuta FDAARGOS_1026	GCF_016127655.1	Complete
Brevundimonas diminuta KX-1	GCF_022654015.1	Complete
Brevundimonas diminuta NCTC8545	GCF_900445995.1	Reference
Brevundimonas fluminis LA-55	GCF_003934285.1	Reference
Brevundimonas goettingensis LVF2	GCF_017487405.1	Reference and complete

Brevundimonas halotolerans MCS24	GCF_003730275.1	Reference
Brevundimonas huaxiensis 090558	GCF_014218725.1	Reference
Brevundimonas intermedia B-10	GCF_004614235.1	Complete
Brevundimonas lenta DSM 23960	GCF_014196335.1	Reference
Brevundimonas lutea NS26	GCF_003704105.1	Reference
Brevundimonas mediterranea D151-2-6	GCF_011064825.1	Complete
Brevundimonas mediterranea DSM 14878	GCF_014196125.1	Reference
Brevundimonas naejangsanensis B1	GCF_000635915.2	Complete
Brevundimonas naejangsanensis BRV3	GCF_003627995.1	Complete
Brevundimonas naejangsanensis DSM 23858	GCF_000421705.1	Reference
Brevundimonas naejangsanensis FS1091	GCF_004421065.1	Complete
Brevundimonas nasdae A30	GCF_019395165.1	Complete
Brevundimonas nasdae Au29	GCF_019395145.1	Reference and complete
Brevundimonas pishanensis CHPC 1.3453	GCF_022750635.1	Reference
Brevundimonas pondensis LVF1	GCF_020524625.1	Reference and complete
Brevundimonas poindexterae EaN21-13	GCF_017487345.1	Reference
Brevundimonas sp. AJA228-03	GCF_017795885.1	Complete
Brevundimonas sp. SSC2	GCA_013693835.1	Complete
Brevundimonas sp. Bb-A	GCF_009394735.1	Complete
Brevundimonas sp. CS1	GCF_017086445.1	Complete
Brevundimonas sp. DS20	GCF_001310255.1	Complete
Brevundimonas sp. GW460-12-10-14-LB2	GCF_001636925.1	Complete
Brevundimonas sp. LM2	GCF_002002865.1	Complete
Brevundimonas sp. M20	GCF_006547065.1	Complete
Brevundimonas sp. MF30-B	GCF_004683885.1	Complete
Brevundimonas sp. PAMC22021	GCF_019443405.1	Complete
Brevundimonas sp. 'scallop'	GCF_011045535.1	Complete
Brevundimonas sp. SGAir0440	GCF_005484585.1	Complete
Brevundimonas subvibrioides ATCC 15264	GCF_000144605.1	Reference and complete
Brevundimonas terrae DSM 17329	GCF_011761985.1	Reference
Brevundimonas vancanneytii NCTC9239	GCF_901421975.1	Complete
Brevundimonas variabilis DSM 4737	GCF_014199945.1	Reference
Brevundimonas vesicularis FDAARGOS 289	GCF_002208825.2	Reference and complete
Brevundimonas viscosa CGMCC 1.10683	GCF_900116065.1	Reference
Brevundimonas vitisensis GR-TSA-9	GCF_016656965.1	Reference and complete

Table S2. 16S rRNA sequences used to assemble the 16S rRNA phylogenetic tree.

Strain	NCBI Reference Sequence	Size
Brevundimonas subvibrioides ATCC 15264	NR_074136.1	1449 bp
Brevundimonas viscosa strain F3	NR_117900.2	1422 bp
Brevundimonas olei strain MJ15	NR_117268.1	1401 bp
Brevundimonas diminuta ATCC 11568	NR_117188.1	1334 bp
Brevundimonas balnearis strain FDRGB2b	NR_156861.1	1399 bp
Brevundimonas canariensis strain GTAE24	NR_156857.1	1431 bp
Brevundimonas albigilva strain NHI-13	NR_148791.1	1442 bp
Brevundimonas naejangsanensis strain BIO-TAS2-2	NR_116722.1	1418 bp
Brevundimonas basaltis strain J22	NR_116194.1	1339 bp
Brevundimonas staleyi strain FWC43	NR_114710.1	1416 bp
Brevundimonas poindexterae strain FWC40	NR_114709.1	1418 bp
Brevundimonas abyssalis TAR-001	NR_114308.1	1406 bp
Brevundimonas aveniformis strain EMB102	NR_043770.1	1403 bp
Brevundimonas subvibrioides ATCC 15264 strain CB81	NR_037107.1	1416 bp
Brevundimonas faecalis strain CS20.3	NR_117187.1	1323 bp
Brevundimonas subvibrioides ATCC 15264	NR_112028.1	1239 bp
Brevundimonas diminuta ATCC 11568	NR_040805.1	1452 bp
Brevundimonas lutea strain NS26	NR_171448.1	1389 bp
Brevundimonas humi strain CA-15	NR_159912.1	1409 bp
Brevundimonas denitrificans strain TAR-002	NR_133989.1	1385 bp
Brevundimonas lenta strain DS-18	NR_044186.1	1418 bp
Brevundimonas terrae strain KSL-145	NR_043726.1	1418 bp
Brevundimonas kwangchunensis strain KSL-102	NR_043315.1	1416 bp
Brevundimonas intermedia strain CIP 106444	NR_116137.1	1260 bp
Brevundimonas vesicularis strain CIP 101035	NR_116136.1	1260 bp
Brevundimonas bacteroides strain CIP 101031	NR_116135.1	1260 bp
Brevundimonas diminuta strain LMG 2089	NR_114708.1	1418 bp
Brevundimonas intermedia strain ATCC 15262	NR_041966.1	1416 bp
Brevundimonas alba strain DSM 4736	NR_041965.1	1417 bp
Brevundimonas mediterranea strain V4.BO.10	NR_037108.1	1416 bp
Brevundimonas variabilis strain CB17	NR_037106.1	1416 bp

Brevundimonas bacteroides strain CB7	NR_037105.1	1416 bp
Brevundimonas vesicularis strain Busing	NR_037104.1	1416 bp
Brevundimonas aurantiaca strain CB-R	NR_028889.1	1416 bp
Brevundimonas nasdae strain W1-2B	NR_028633.1	1427 bp
Brevundimonas halotolerans strain MCS24	NR_118765.1	1292 bp
Brevundimonas subvibrioides strain NBRC 16000	NR_113834.1	1386 bp
Brevundimonas bullata strain NBRC 13290	NR_113611.1	1389 bp
Brevundimonas diminuta strain NBRC 12697	NR_113602.1	1388 bp
Brevundimonas vesicularis strain NBRC 12165	NR_113586.1	1386 bp
Brevundimonas diminuta strain JCM 2788	NR_113238.1	1385 bp
Brevundimonas vesicularis strain IAM 12105	NR_112078.1	1450 bp
Brevundimonas bacteroides strain ATCC 15254	NR_112031.1	1214 bp
Brevundimonas bullata strain IAM 13153	NR_025831.1	1415 bp
Brevundimonas sp.	-	1459 bp
Henriciella pelagia strain LA220	NR_157792.1	1442 bp

Table S3. *Brevundimonas* ssp. used in MLSA analysis along with its accession number and annotation type.

Species	Acession numbers	Annotation Name
Brevundimonas abyssalis TAR-001*	GCF_000466985.1	NCBI Prokaryotic Genome Annotation Pipeline (PGAP)
Brevundimonas alba DSM 4736*	GCF_011927945.1	NCBI Prokaryotic Genome Annotation Pipeline (PGAP)
Brevundimonas aurantiaca FXH-172*	GCF_020782355.1	NCBI Prokaryotic Genome Annotation Pipeline (PGAP)
Brevundimonas albigilva T7	GCF_023573565.1	NCBI Prokaryotic Genome Annotation Pipeline (PGAP)
Brevundimonas aveniformis DSM 17977*	GCF_000428765.1	NCBI Prokaryotic Genome Annotation Pipeline (PGAP)
Brevundimonas bacteroides DSM 4726*	GCF_000701445.1	NCBI Prokaryotic Genome Annotation Pipeline (PGAP)
Brevundimonas basaltis DSM 25335*	GCF_014202075.1	NCBI Prokaryotic Genome Annotation Pipeline (PGAP)
Brevundimonas bullata HAMBI_262*	GCF_003350205.1	NCBI Prokaryotic Genome Annotation Pipeline (PGAP)
Brevundimonas diminuta ATCC(B) 19146	GCF_004102925.1	NCBI Prokaryotic Genome Annotation Pipeline (PGAP)

Brevundimonas diminuta FDAARGOS_1026	GCF_016127655.1	NCBI Prokaryotic Genome Annotation Pipeline (PGAP)
Brevundimonas diminuta NCTC8545*	GCF_900445995.1	NCBI Prokaryotic Genome Annotation Pipeline (PGAP)
Brevundimonas fluminis LA-55*	GCF_003934285.1	NCBI Prokaryotic Genome Annotation Pipeline (PGAP)
Brevundimonas goettingensis LVF2*	GCF_017487405.1	NCBI Prokaryotic Genome Annotation Pipeline (PGAP)
Brevundimonas halotolerans MCS24*	GCF_003730275.1	NCBI Prokaryotic Genome Annotation Pipeline (PGAP)
Brevundimonas huaxiensis 090558*	GCF_014218725.1	NCBI Prokaryotic Genome Annotation Pipeline (PGAP)
Brevundimonas intermedia B-10	GCF_004614235.1	NCBI Prokaryotic Genome Annotation Pipeline (PGAP)
Brevundimonas lenta DSM 23960*	GCF_014196335.1	NCBI Prokaryotic Genome Annotation Pipeline (PGAP)
Brevundimonas lutea NS26*	GCF_003704105.1	NCBI Prokaryotic Genome Annotation Pipeline (PGAP)
Brevundimonas mediterranea D151-2-6	GCF_011064825.1	NCBI Prokaryotic Genome Annotation Pipeline (PGAP)
Brevundimonas mediterranea DSM 14878 *	GCF_014196125.1	NCBI Prokaryotic Genome Annotation Pipeline (PGAP)
Brevundimonas naejangsanensis B1	GCF_000635915.2	NCBI Prokaryotic Genome Annotation Pipeline (PGAP)
Brevundimonas naejangsanensis BRV3	GCF_003627995.1	NCBI Prokaryotic Genome Annotation Pipeline (PGAP)
Brevundimonas naejangsanensis DSM 23858*	GCF_000421705.1	NCBI Prokaryotic Genome Annotation Pipeline (PGAP)
Brevundimonas naejangsanensis FS1091	GCF_004421065.1	NCBI Prokaryotic Genome Annotation Pipeline (PGAP)
Brevundimonas nasdae A30	GCF_019395165.1	NCBI Prokaryotic Genome Annotation Pipeline (PGAP)
Brevundimonas nasdae Au29*	GCF_019395145.1	NCBI Prokaryotic Genome Annotation Pipeline (PGAP)
Brevundimonas pishanensis CHPC 1.3453*	GCF_022750635.1	NCBI Prokaryotic Genome Annotation Pipeline (PGAP)

Brevundimonas pondensis LVF1*	GCF_020524625.1	NCBI Prokaryotic Genome Annotation Pipeline (PGAP)
Brevundimonas poindexterae EaN21-13*	GCF_017487345.1	NCBI Prokaryotic Genome Annotation Pipeline (PGAP)
Brevundimonas sp. AJA228-03	GCF_017795885.1	NCBI Prokaryotic Genome Annotation Pipeline (PGAP)
Brevundimonas sp. SSC2	GCA_013693835.1	NCBI Prokaryotic Genome Annotation Pipeline (PGAP)
Brevundimonas sp. Bb-A	GCF_009394735.1	NCBI Prokaryotic Genome Annotation Pipeline (PGAP)
Brevundimonas sp. CS1	GCF_017086445.1	NCBI Prokaryotic Genome Annotation Pipeline (PGAP)
Brevundimonas sp. M20	GCF_006547065.1	NCBI Prokaryotic Genome Annotation Pipeline (PGAP)
Brevundimonas sp. MF30-B	GCF_004683885.1	NCBI Prokaryotic Genome Annotation Pipeline (PGAP)
Brevundimonas sp. PAMC22021	GCF_019443405.1	NCBI Prokaryotic Genome Annotation Pipeline (PGAP)
Brevundimonas sp. 'scallop'	GCF_011045535.1	NCBI Prokaryotic Genome Annotation Pipeline (PGAP)
Brevundimonas sp. SGAir0440	GCF_005484585.1	NCBI Prokaryotic Genome Annotation Pipeline (PGAP)
Brevundimonas terrae DSM 17329*	GCF_011761985.1	NCBI Prokaryotic Genome Annotation Pipeline (PGAP)
Brevundimonas vancanneytii NCTC9239	GCF_901421975.1	NCBI Prokaryotic Genome Annotation Pipeline (PGAP)
Brevundimonas variabilis DSM 4737*	GCF_014199945.1	NCBI Prokaryotic Genome Annotation Pipeline (PGAP)
Brevundimonas vesicularis FDAARGOS 289*	GCF_002208825.2	NCBI Prokaryotic Genome Annotation Pipeline (PGAP)
Brevundimonas viscosa CGMCC 1.10683*	GCF_900116065.1	NCBI Prokaryotic Genome Annotation Pipeline (PGAP)
Brevundimonas vitisensis GR-TSA-9*	GCF_016656965.1	NCBI Prokaryotic Genome Annotation Pipeline (PGAP)

* Reference genomes *Brevundimonas*

Table S4. *Breuvdimonas* ssp. used in the ANI and dDDH calculation analysis with their respective accession numbers.

**Species**	**Accession numbers**
Brevundimonas abyssalis TAR-001*	GCF_000466985.1
Brevundimonas alba DSM 4736*	GCF_011927945.1
Brevundimonas aurantiaca FXH-172*	GCF_020782355.1
Brevundimonas albigilva T7	GCF_023573565.1
Brevundimonas aveniformis DSM 17977*	GCF_000428765.1
Brevundimonas bacteroides DSM 4726*	GCF_000701445.1
Brevundimonas basaltis DSM 25335*	GCF_014202075.1
Brevundimonas bullata HAMBI_262*	GCF_003350205.1
Brevundimonas diminuta ATCC(B) 19146	GCF_004102925.1
Brevundimonas diminuta FDAARGOS_1026	GCF_016127655.1
Brevundimonas diminuta KX-1	GCF_022654015.1
Brevundimonas diminuta NCTC8545*	GCF_900445995.1
Brevundimonas fluminis LA-55*	GCF_003934285.1
Brevundimonas goettingensis LVF2*	GCF_017487405.1
Brevundimonas halotolerans MCS24*	GCF_003730275.1
Brevundimonas huaxiensis 090558*	GCF_014218725.1
Brevundimonas intermedia B-10	GCF_004614235.1
Brevundimonas lenta DSM 23960*	GCF_014196335.1
Brevundimonas lutea NS26*	GCF_003704105.1
Brevundimonas mediterranea D151-2-6	GCF_011064825.1
Brevundimonas mediterranea DSM 14878 *	GCF_014196125.1
Brevundimonas naejangsanensis B1	GCF_000635915.2
Brevundimonas naejangsanensis BRV3	GCF_003627995.1
Brevundimonas naejangsanensis DSM 23858*	GCF_000421705.1
Brevundimonas naejangsanensis FS1091	GCF_004421065.1
Brevundimonas nasdae A30	GCF_019395165.1
Brevundimonas nasdae Au29*	GCF_019395145.1
Brevundimonas pishanensis CHPC 1.3453*	GCF_022750635.1
Brevundimonas pondensis LVF1*	GCF_020524625.1
Brevundimonas poindexterae EaN21-13*	GCF_017487345.1
Brevundimonas sp. AJA228-03	GCF_017795885.1
Brevundimonas sp. SSC2	GCA_013693835.1
Brevundimonas sp. Bb-A	GCF_009394735.1
Brevundimonas sp. CS1	GCF_017086445.1

Brevundimonas sp. DS20	GCF_001310255.1
Brevundimonas sp. GW460-12-10-14-LB2	GCF_001636925.1
Brevundimonas sp. LM2	GCF_002002865.1
Brevundimonas sp. M20	GCF_006547065.1
Brevundimonas sp. MF30-B	GCF_004683885.1
Brevundimonas sp. PAMC22021	GCF_019443405.1
Brevundimonas sp. 'scallop'	GCF_011045535.1
Brevundimonas sp. SGAir0440	GCF_005484585.1
Brevundimonas subvibrioides ATCC 15264*	GCF_000144605.1
Brevundimonas terrae DSM 17329*	GCF_011761985.1
Brevundimonas vancanneytii NCTC9239	GCF_901421975.1
Brevundimonas variabilis DSM 4737*	GCF_014199945.1
Brevundimonas vesicularis FDAARGOS 289*	GCF_002208825.2
Brevundimonas viscosa CGMCC 1.10683*	GCF_900116065.1
Brevundimonas vitisensis GR-TSA-9*	GCF_016656965.1

* Reference genomes *Brevundimonas*

Table S5. Reference genomes used in TYGS analysis and their respective accession numbers.

**Species**	**Accession numbers**
Brevundimonas abyssalis TAR-001	GCF_000466985.1
Brevundimonas alba DSM 4736	GCF_011927945.1
Brevundimonas aurantiaca FXH-172	GCF_020782355.1
Brevundimonas bacteroides DSM 4726	GCF_000701445.1
Brevundimonas basaltis DSM 25335	GCF_014202075.1
Brevundimonas bullata HAMBI_262	GCF_003350205.1
Brevundimonas diminuta NCTC8545	GCF_900445995.1
Brevundimonas fluminis LA-55	GCF_003934285.1
Brevundimonas goettingensis LVF2	GCF_017487405.1
Brevundimonas huaxiensis 090558	GCF_014218725.1
Brevundimonas lenta DSM 23960	GCF_014196335.1
Brevundimonas lutea NS26	GCF_003704105.1
Brevundimonas naejangsanensis DSM 23858	GCF_000421705.1
Brevundimonas nasdae Au29	GCF_019395145.1
Brevundimonas pishanensis CHPC 1.3453	GCF_022750635.1
Brevundimonas subvibrioides ATCC 15264	GCF_000144605.1
Brevundimonas vesicularis FDAARGOS 289	GCF_002208825.2
Brevundimonas viscosa CGMCC 1.10683	GCF_900116065.1
Brevundimonas vitisensis GR-TSA-9	GCF_016656965.1

Table S6. *Brevundimonas* genomes used in the core and accessory genome analysis.

Species	Accession numbers
Brevundimonas abyssalis TAR-001*	GCF_000466985.1
Brevundimonas alba DSM 4736*	GCF_011927945.1
Brevundimonas aurantiaca FXH-172*	GCF_020782355.1
Brevundimonas albigilva T7	GCF_023573565.1
Brevundimonas aveniformis DSM 17977*	GCF_000428765.1
Brevundimonas bacteroides DSM 4726*	GCF_000701445.1
Brevundimonas basaltis DSM 25335*	GCF_014202075.1
Brevundimonas bullata HAMBI_262*	GCF_003350205.1
Brevundimonas diminuta ATCC(B) 19146	GCF_004102925.1
Brevundimonas diminuta FDAARGOS_1026	GCF_016127655.1
Brevundimonas diminuta KX-1	GCF_022654015.1
Brevundimonas diminuta NCTC8545*	GCF_900445995.1
Brevundimonas fluminis LA-55*	GCF_003934285.1
Brevundimonas goettingensis LVF2*	GCF_017487405.1
Brevundimonas halotolerans MCS24*	GCF_003730275.1
Brevundimonas huaxiensis 090558*	GCF_014218725.1
Brevundimonas intermedia B-10	GCF_004614235.1
Brevundimonas lenta DSM 23960*	GCF_014196335.1
Brevundimonas lutea NS26*	GCF_003704105.1
Brevundimonas mediterranea D151-2-6	GCF_011064825.1
Brevundimonas mediterranea DSM 14878 *	GCF_014196125.1
Brevundimonas naejangsanensis B1	GCF_000635915.2
Brevundimonas naejangsanensis BRV3	GCF_003627995.1
Brevundimonas naejangsanensis DSM 23858*	GCF_000421705.1
Brevundimonas naejangsanensis FS1091	GCF_004421065.1
Brevundimonas nasdae A30	GCF_019395165.1
Brevundimonas nasdae Au29*	GCF_019395145.1
Brevundimonas pishanensis CHPC 1.3453*	GCF_022750635.1
Brevundimonas pondensis LVF1*	GCF_020524625.1
Brevundimonas poindexterae EaN21-13*	GCF_017487345.1
Brevundimonas sp. AJA228-03	GCF_017795885.1
Brevundimonas sp. SSC2	GCA_013693835.1
Brevundimonas sp. Bb-A	GCF_009394735.1

Brevundimonas sp. CS1	GCF_017086445.1
Brevundimonas sp. DS20	GCF_001310255.1
Brevundimonas sp. GW460-12-10-14-LB2	GCF_001636925.1
Brevundimonas sp. LM2	GCF_002002865.1
Brevundimonas sp. M20	GCF_006547065.1
Brevundimonas sp. MF30-B	GCF_004683885.1
Brevundimonas sp. PAMC22021	GCF_019443405.1
Brevundimonas sp. 'scallop'	GCF_011045535.1
Brevundimonas sp. SGAir0440	GCF_005484585.1
Brevundimonas subvibrioides ATCC 15264*	GCF_000144605.1
Brevundimonas terrae DSM 17329*	GCF_011761985.1
Brevundimonas vancanneytii NCTC9239	GCF_901421975.1
Brevundimonas variabilis DSM 4737*	GCF_014199945.1
Brevundimonas vesicularis FDAARGOS 289*	GCF_002208825.2
Brevundimonas viscosa CGMCC 1.10683*	GCF_900116065.1
Brevundimonas vitisensis GR-TSA-9*	GCF_016656965.1

* Reference genomes *Brevundimonas*

References

1. Minarini LAR, Lucia A, Darini C. 2012. Mutations in the Quinolone Resistance Determining-Regions of *gyrA* and *parC* in Enterobacteriaceae Isolates from Brazil. Brazilian Journal of Microbiology 1309–1314.
2. Weigel LM, Steward CD, Tenover FC. 1998. *gyrA* Mutations Associated with Fluoroquinolone Resistance in Eight Species of *Enterobacteriaceae*. Antimicrob Agents Chemother 42:2661–2667.
3. Han XY, Andrade RA. 2005. Brevundimonas diminuta infections and its resistance to fluoroquinolones. Journal of Antimicrobial Chemotherapy 55:853–859.
4. Pitt ME, Cao MD, Butler MS, Ramu S, Ganesamoorthy D, Blaskovich MAT, Coin LJM, Cooper MA. 2019. Octapeptin C4 and polymyxin resistance occur via distinct pathways in an epidemic XDR Klebsiella pneumoniae ST258 isolate. Journal of Antimicrobial Chemotherapy 74:582–593.
5. Berglund B. 2019. Acquired Resistance to Colistin via Chromosomal And Plasmid-Mediated Mechanisms in Klebsiella pneumoniae. Infectious Microbes and Diseases 1:10–19.
